# The performance of coalescent-based species tree estimation methods under models of missing data

**DOI:** 10.1186/s12864-018-4619-8

**Published:** 2018-05-08

**Authors:** Michael Nute, Jed Chou, Erin K. Molloy, Tandy Warnow

**Affiliations:** 1 Department of Statistics, University of Illinois at Urbana-Champaign, 725 S. Wright St., Champaign, IL, 61820 USA; 2 Department of Mathematics, University of Illinois at Urbana-Champaign, 1409 W. Green St., Urbana, IL, 61801 USA; 3 Department of Computer Science, University of Illinois at Urbana-Champaign, 201 North Goodwin Avenue, Urbana, IL, 61801 USA

**Keywords:** Species tree, Multi-species coalescent, Missing data, Incomplete lineage sorting, ASTRAL, ASTRID, MP-EST, SVDquartets

## Abstract

**Background:**

Estimation of species trees from multiple genes is complicated by processes such as incomplete lineage sorting, gene duplication and loss, and horizontal gene transfer, that result in gene trees that differ from each other and from the species phylogeny. Methods to estimate species trees in the presence of gene tree discord due to incomplete lineage sorting have been developed and proved to be statistically consistent when gene tree discord is due only to incomplete lineage sorting and every gene tree includes the full set of species.

**Results:**

We establish statistical consistency of certain coalescent-based species tree estimation methods under some models of taxon deletion from genes. We also evaluate the impact of missing data on four species tree estimation methods (ASTRAL-II, ASTRID, MP-EST, and SVDquartets) using simulated datasets with varying levels of incomplete lineage sorting, gene tree estimation error, and degrees/patterns of missing data.

**Conclusions:**

All the species tree estimation methods improved in accuracy as the number of genes increased and often produced highly accurate species trees even when the amount of missing data was large. These results together indicate that accurate species tree estimation is possible under a variety of conditions, even when there are substantial amounts of missing data.

**Electronic supplementary material:**

The online version of this article (10.1186/s12864-018-4619-8) contains supplementary material, which is available to authorized users.

## Background

The estimation of a species phylogeny from multiple loci is confounded by biological processes, such as horizontal gene transfer and incomplete lineage sorting, that cause individual gene tree topologies to differ from each other [1]. While some of these processes require phylogenetic networks for proper modeling of the species phylogeny, other processes, including incomplete lineage sorting (ILS) and gene duplication and loss, are still consistent with a species tree. Estimating species trees in the presence of gene tree heterogeneity is challenging.

ILS, which is modeled by the well-studied multi-species coalescent (MSC) model [1–3], is considered to be a major cause for this discordance [4], and the subject of substantial study in recent years. Many methods have been developed to estimate the species tree in the presence of ILS in a statistically consistent manner, which means that as the amount of data increases, the unrooted species tree topology estimated by the method converges in probability to the true unrooted species tree topology. Examples of methods for species tree estimation that are statistically consistent under the MSC model include ASTRAL-I [5] and its improved version ASTRAL-II [6], ASTRID [7], *BEAST [8], BEST [9], the population tree in BUCKy [10], GLASS [11], METAL [12], MP-EST [13], NJst [14], SMRT [15], SNAPP [16], STAR [17], STEAC [17], and STEM [18]. Some of these methods (e.g., ASTRAL-I, ASTRID, BUCKy-pop, and NJst) estimate just the species tree topology but not the branch lengths in coalescent units, while others (e.g., ASTRAL-II, BEST, *BEAST, and MP-EST) also estimate the branch lengths. In this paper, we will refer to all methods that have been proven to be statistically consistent under the MSC model as “coalescent-based” species tree estimation methods.

One of the key assumptions in the proofs of statistical consistency for standard methods is that every gene is present in every species. This assumption is unrealistic for many empirical datasets (e.g., the plant transcriptome dataset studied in [19], the landfowl ultra-conserved elements (UCE) dataset studied in [20], and the iguanian lizard UCE dataset studied in [21]), where many genes are not present for some species (and so are “incomplete”). There are multiple causes for incomplete genes (more commonly referred to as “missing data”). For example, while some genes are universal, others have more recent origins and so may only occupy certain clades in the tree. Genes can also be lost (as modeled by gene birth-death or duplication/loss scenarios). The selection of primers used to assemble the datasets can fail to detect some genes in distantly related species, or whole genomes can have poorly assembled regions if coverage is insufficient; these factors can result in the gene not being included in the assembled data for a particular species. Furthermore, some species trees are constructed by taking gene trees from prior publications, which can have somewhat different species sets. For these and other reasons, missing data occur frequently in large-scale phylogenomic datasets (as discussed in [21–24]). Therefore, the statistical properties of species tree estimation methods when not all genes are present in all the species are important methodological considerations in phylogenomics. The most basic of these questions is whether a method remains statistically consistent when gene trees can be incomplete. Yet, it is unknown whether coalescent-based species tree estimation methods are statistically consistent under the MSC model in the presence of missing data.

## Methods

This study evaluates the statistical consistency of species tree estimation methods under two models of taxon deletion, and also provides an experimental evaluation of several leading methods on simulated datasets where taxa are deleted under these two models.

### Theoretical framework

We explore the statistical consistency of species tree estimation methods under two models of taxon deletion. The first is a simple *i.i.d.* model of missing data (denoted *M*_*iid*_) where every species is missing from every gene with the same probability *p*>0. The second is a more general class of models where, for some constant *k*, each subset of *k* species has non-zero probability of being present in the data for a randomly selected gene. We refer to this as the “full subset coverage” model (denoted *M*_*fsc*_). The *M*_*fsc*_ model includes the simpler *i.i.d.* model as a special case but also includes the models of taxon deletion considered in [7, 23–25]. In this study, we focus on coalescent-based species tree methods that compute summary statistics for subsets of the taxon set and then use those summary statistics to estimate the species tree. We show that whenever these calculated summary statistics are not impacted by deleting species outside the subset of interest, then the coalescent-based species tree estimation method will be statistically consistent under models of taxon deletion. Taxon-deletion models under which coalescent-based species tree estimation methods cannot be statistically consistent are also discussed.

**Multi-Species Coalescent** The multi-species coalescent is a population genetics model that describes the evolution of individual genes within a population-level species tree [26]. Specifically, a species tree $\mathcal {T}=(T,\Theta)$ with topology *T* and branch lengths *Θ* is given (but unknown) on a set of *n* taxa, $\mathcal {X}=\{x_{i}\}_{i=1}^{n}$, where the branch lengths are denominated in “coalescent units”. In models with a population size parameter and time between speciation events, coalescent units are units of time that are normalized by population size. This species tree then parameterizes a probability density function for a random variable $G(\mathcal {T})$ defined over all possible gene trees on $\mathcal {X}$.

The generation of a random gene tree occurs by modelling the time since ancestral coalescence as though it were a forward-time Markov process: at every leaf of the species tree $\mathcal {T}$ a lineage begins and grows backward in time. As each lineage grows, it will eventually reach a speciation event in the species tree. At that point, it enters a common population with the lineage(s) from the neighboring branch and they continue to grow backward in time as distinct lineages. Once they are in a common population they are eligible to coalesce into a common lineage, and that happens according to a Poisson process with constant hazard rate *λ*. Namely, for any pair of lineages *i* and *j* in a common population, the time *τ*_*ij*_ until their ancestral coalescence has an exponential distribution:

$$\tau_{ij}\sim f_{\lambda}(\tau_{ij})=\lambda e^{-\lambda \tau_{ij}} $$

Likewise, in a population with *k* distinct, uncoalesced lineages, the time until the next coalescent event is the first one out of the ${{k}\choose {2}}$ pairwise processes, and thus also has exponential distribution with rate ${{k}\choose {2}}\lambda $:

$$\tau_{k\rightarrow k-1}\sim f(\tau)=\frac{k(k-1)}{2}\lambda e^{-\frac{k(k-1)}{2}\lambda \tau} $$

In this way, the lineages grow and coalesce until all lineages have coalesced into a single one, at which point the process is complete and the lineages have formed a binary gene tree.

For a gene tree $g\sim G(\mathcal {T})$, an additional assumption can be made regarding a sequence evolution model that may generate a set of sequences $s_{g}=\left \{s_{g1},\dots,s_{gn}\right \}$ (one sequence for each taxon in $\mathcal {X}$). Let the leaf set of gene tree *g* be denoted as $\mathcal {L}(g)$. Given a collection of genes $g_{1},\dots,g_{m} \sim G(\mathcal {T})$, the coalescent-based species tree estimation problem is the challenge of estimating the species tree topology *T* (typically in its unrooted form) from the input data, which may include the gene trees (usually unrooted), the accompanying sequences, or both.

Thus, coalescent-based species tree estimation methods can work with a variety of different types of inputs. Usually such methods assume that the estimation of gene trees given sequence data can be done in a statistically consistent manner, which is true in the case of the most common models [27]. In this paper, we will consider the input data *I* to include, broadly, the gene trees themselves (one per gene) with or without branch lengths, or the multiple sequence alignments (one per gene), or both, depending on the method. In either case, it is natural to consider the input data *I* as being potentially restricted to a subset $\mathcal {X}'$ of the taxa by considering, respectively, the subtrees of each gene tree restricted to the leaves corresponding to taxa in $\mathcal {X}'$, or the multiple sequence alignment of only the sequences corresponding to taxa in $\mathcal {X}'$. We will refer occasionally to this restricted data as $I|_{\mathcal {X}'}$. In contexts where the number of genes may vary and is indexed by *m*, the input data *I* on *m* genes are correspondingly indexed as *I*_*m*_.

In what follows, all trees (whether gene trees or species trees) are assumed to be binary (i.e., fully resolved) trees with leaves labeled by elements from a set $\mathcal {X}$ of species.

**Tuple-based methods** We will establish properties about statistical consistency in the presence of missing data for a class of coalescent-based species tree estimation methods that we collectively refer to as “tuple-based methods”. Many coalescent-based species tree estimation methods that have been proven to be statistically consistent under the MSC model are tuple-based.

A coalescent-based method is said to be “tuple-based” if there is some $\ell \in \mathbb {Z}_{\geq 2}$ such that the method operates by computing a set of real-valued summary statistics from the input *I* for every subset of *ℓ* species, and then uses these summary statistics (and no other information) to compute the species tree. Furthermore a tuple-based method is called an *ℓ*-tuple-based method (or more simply an *ℓ*-tuple method) to reflect the specific value of *ℓ* on which it bases its summary statistics. We write each tuple-based method as a pair (*F*,*α*), with *F* the function that computes the set of summary statistics from *I* and *α* the function that computes a species tree given *F*(*I*). Also, the set of summary statistics computed by an *ℓ*-tuple method includes one statistic for every tree topology (possibly rooted) on every subset of *ℓ* species.

Since a “tree” on two species is just a path, the 2-tuple methods compute pairwise distances for every pair of species. Examples of 2-tuple methods include NJst and ASTRID, which operate by computing the “average internode distance” between every pair of species. Other 2-tuple methods include GLASS [11] and its variants (e.g., [28]), METAL [12], STAR [17], and STEAC [17], which also compute pairwise distances between every pair of species but use a different technique to do the calculation. 2-tuple methods then compute a tree on the matrix of pairwise distances, using methods such as Neighbor Joining (NJ) [29] or FastME [30]; thus, NJ and FastME serve as the function *α* in the 2-tuple method.

MP-EST and SMRT are 3-tuple methods. MP-EST requires rooted gene trees (and so depends on the strict molecular clock when used with sequence data), and uses the frequency of each rooted 3-leaf tree *t* induced in the input set of gene trees as the summary statistic for *t*. It then seeks the model species tree (topology and branch lengths) that is most likely to produce the observed distribution of rooted 3-leaf gene tree frequencies. SMRT is a site-based method that estimates rooted 3-leaf trees from the concatenated gene sequence alignments, and so depends on the strict molecular clock. SMRT then combines the rooted 3-leaf trees into a tree on the full set of taxa using the modified mincut supertree algorithm [31]. SMRT can be seen as a 3-tuple method by assigning support of 1 to the rooted 3-leaf trees it computes and assigning support of 0 to all other 3-leaf trees.

In contrast to 3-tuple methods (e.g., MP-EST and SMRT), 4-tuple methods operate on unrooted gene trees. For example, ASTRAL-1 [5] and ASTRAL-2 [6] are 4-tuple methods that use the frequency of quartet tree *t* induced in the input gene trees as the real-valued support for *t* and combines these quartet trees into a species tree using a quartet amalgamation method. The population tree in BUCKy [10] (called BUCKy-pop in [32]) computes a single quartet tree for every four leaves using Bayesian concordance factors, and then combines those quartet trees into a species tree. BUCKy-pop is statistically consistent under the MSC model if there are no missing data, and can be seen as a 4-tuple method by using 1 or 0 as the real-valued support for each quartet tree (i.e., 1 for the quartet trees it computes, and 0 for all others). In other words, ASTRAL and BUCKY-pop compute a species tree by applying some quartet amalgamation method to the set of quartet trees, weighted by their support values. For these 4-tuple methods, *α* is the quartet amalgamation technique used to construct the species tree topology *T*.

The number of summary statistics that each type of method computes depends on the value of *ℓ* and the number *n* of species: 2-tuple methods compute $n \choose 2$ summary statistics (one for each pair of species), 3-tuple methods compute $3 {n \choose 3}$ summary statistics (one for each rooted 3-leaf tree), and 4-tuple methods compute $3 {n \choose 4}$ summary statistics (one for each unrooted 4-leaf tree).

The proofs of statistical consistency for tuple-based methods have the following basic steps: first, they show that as the number *m* of genes increases, the vector of summary statistics computed by *F* on input data *I*_*m*_ converges in probability to a constant vector (which we will refer to as *F*_0_). Second, they show that *α*(*F*_0_)=*T*, where *T* is the topology of the true species tree. Third, they show that there is some *δ*>0 so that whenever $L_{\infty }(F_{1},F_{0}) < \delta $ then *α*(*F*_1_)=*α*(*F*_0_)=*T* (here $L_{\infty }$ is the infinity-norm, i.e. the maximum absolute difference of individual vector components). It follows that the algorithm *A*=(*F*,*α*) is statistically consistent under the MSC. Therefore, when we refer to a statistically consistent *ℓ*-tuple method, we will assume that these properties hold for the method when there are no missing data, and then study the impact of missing data on the method.

Proofs of statistical consistency for many coalescent-based methods typically require several extra conditions. For example, the current proofs of statistical consistency of MP-EST, STEM, STAR, and SMRT implicitly or explicitly assume that sequences evolve under the strict molecular clock. Similarly, the proofs of statistical consistency for nearly all methods that operate by combining gene trees require completely correct gene trees (see [33] for an exception to this rule), and it is unknown whether any standard coalescent-based methods that estimate species trees by combining gene trees are statistically consistent in the presence of gene tree estimation error [33]. Another complication in the proofs of statistical consistency is the typical requirement that *α* provide an exact solution to an optimization problem (e.g., finding the species tree that maximizes some optimization criterion with respect to the input gene data). This is generally not an issue for 2-tuple methods, which use methods like neighbor joining [29] to compute trees from distance matrices, but can be a problem for 3-tuple and 4-tuple methods. For example, 4-tuple methods tend to have two steps, where the first step computes a set of quartet trees (using *F*) and the second step computes a tree from the set of quartet trees using *α*. Since quartet tree compatibility is NP-hard [34], quartet amalgamation methods are typically heuristics that have no guarantees (the dynamic programming algorithms in ASTRAL and [35] are two of the few exceptions to this), and may not even be guaranteed to return a tree *T* when given its set of quartet trees. Thus, statistical consistency of coalescent-based methods is complicated, even when there are no missing data.

### Experimental study

We present an empirical study using a large collection of simulated datasets and four species tree estimation methods: ASTRAL-II, ASTRID, MP-EST, and SVDquartets. Three of these (ASTRAL-II, ASTRID, and MP-EST) are “summary methods” (i.e., methods that compute species trees by combining gene trees) that are statistically consistent under the MSC model when there are no missing data. The fourth is SVDquartets, a popular “site-based” method that computes the species tree directly from gene sequence alignments, but which has not yet been proven to be statistically consistent.

This simulation study examines how two different models of missing data, the *M*_*iid*_ model and a “clade-based” model (a subfamily of the *M*_*fsc*_ model, and denoted *M*_*clade*_), affect the amount of data required to recover the true species tree with high probability. The selection of these two taxon deletion models reflects conditions that can occur in biological studies. The *M*_*iid*_ model reflects conditions where genes collected from prior studies are used, and each collection of genes can be based on a set of species that may only contain a subset of all species under consideration. The *M*_*iid*_ also addresses the case where gene assembly is poor due to low coverage, so that genes are randomly missing from some genomes. The *M*_*clade*_ model reflects conditions where genes naturally occupy only a clade within the tree, because the gene is “born” at the root of the clade (this is the same as the “SMIDgen” model described in [36]).

**Simulated datasets** The 26-taxon, 1000-gene simulated datasets (without missing data) were obtained from [24]. This collection of datasets included model conditions defined by species tree height (10M, 2M, or 500K) and speciation rate (10^−7^ or 10^−6^). As the population size was fixed across all model conditions, species tree height corresponds to the level of ILS, with shorter species trees having greater levels of ILS. Speciation rates of 10^−7^ and 10^−6^ correspond to deep and recent speciation, respectively. Each of these six model conditions has 20 replicate datasets. The Robinson-Foulds (RF) distance [37] between the true species and the true gene trees averaged over all gene trees (referred to here as the simply the “average distance” or AD) was computed to approximate the level of ILS for different model conditions. For model conditions with deep speciation, the AD averaged across all 20 replicates was 14, 47, and 75% for species tree heights of 10M, 2M, and 500K, respectively. The average AD values under recent speciation were slightly lower and are provided in Additional file 1.

**Generation of missing data** The simulated datasets described above have no missing data; therefore, we deleted taxa from the genes and re-estimated gene trees to create datasets with missing data for this study. We first considered an *M*_*iid*_ model of missing data for which every species is missing from every gene with the same probability *p*>0. Specifically, datasets with *p*=0.30 and *p*=0.60 were created as follows. For each taxon, a vector *p* of length 1000 (the number of genes) was created and populated with random samples drawn from a uniform distribution over the interval [0,1). Entries in the vector that were smaller than 0.30 became 0 (i.e., the taxon was missing and hence deleted from the true gene tree and gene alignment) and otherwise became 1 (i.e., the taxon was present in the true gene tree and gene alignment). This process was simply repeated for *p*=0.60. As expected, the average amount of missing data across all datasets for *p*=0.30 and *p*=0.60 was 30 and 60%, respectively.

**Fig. 1:**
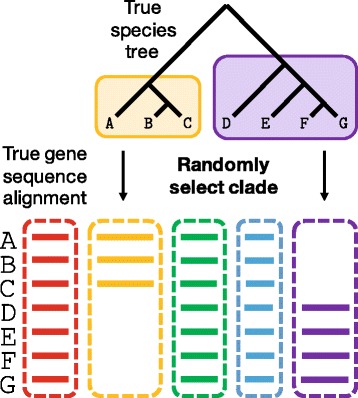
Clade-based missing data. A true species tree on seven species (labeled A-G) and five true gene sequence alignments (colored red, yellow, green, blue, and purple) are shown in this schematic. The red, green, and blue genes have no missing data. The yellow and purple genes have clade-based missing data, created by selecting a clade in the true species tree at random and then deleting taxa outside of the clade from the true gene tree (not shown) and the true gene sequence alignment. For the yellow gene, the clade containing A, B, and C is selected from the true species tree, and hence, species D, E, F, and G are deleted from the yellow gene. Similarly, for the purple gene, the clade containing D, E, F, and G is selected from the true species tree, and hence, species A, B, and C are deleted from the purple gene

We also considered the *M*_*clade*_ model of missing data (Fig. 1). Specifically, we identified clades in the true species tree with between 5 and 24 taxa (out of the original 26 taxa). Then for each of the 1000 genes, one of these previously identified clades was selected at random from a uniform distribution, and taxa outside of this clade were deleted from the gene alignment. Based on the selection of clades, 8 to 80% of taxa were deleted from genes with clade-based missing data, and the mean (± standard deviation) fraction of taxa missing per gene with clade-based missing data was 62±5*%*. Notably, this protocol automatically deleted the outgroup taxon from the gene alignment, so that it was not possible to reconstruct a species tree on the full set of taxa from the resulting gene trees. Therefore, genes both with and without clade-based missing data were sampled to create datasets with varying percentages of incomplete genes. The mean (± standard deviation) percentage of missing data was 34±3 and 59±5 when 55 and 95% of the 1000 genes were incomplete.

**Gene tree estimation** Taxa were deleted from both the true gene trees and the true gene sequence alignments. Gene trees were estimated on alignments with and without missing data using a single run of RAxML v8.2.8 (GTRGAMMA model), and nearly 500,000 gene trees were estimated for this study.

**Species tree estimation** We studied the performance of four methods for species tree estimation: three gene tree summary methods (ASTRAL v4.10.5, ASTRID v1.1, MP-EST v1.5) and one site-based method (SVDquartets using PAUP* v4a154). Species trees were estimated using summary methods on both true and estimated gene trees, specifically taking the first 50, 200, or 1000 genes for each dataset. Of the three summary methods, only MP-EST requires rooted genes. To root each gene tree, we used the outgroup if it was available; otherwise, we used the midpoint of the longest leaf-to-leaf path with Dendropy v4.3.0 [38]. The best pseudo-likelihood scoring species tree was taken from ten independent runs of MP-EST. Gene alignments had been generated without insertions/deletions, and thus SVDquartets was run on the true gene alignments by concatenating the first 50, 200, or 1000 gene sequence alignments for each dataset. SVDquartets completed on all but one dataset (see Additional file 1, for details). All four methods were run in default modes (although see comment above regarding the use of ten independent runs of MP-EST). We report species tree error using the normalized RF error rate.

## Results

We present the theoretical results, establishing statistical consistency (or failure to be statistically consistent), for different species tree estimation methods. We then present the results of our performance study.

### New theoretical results

**Extension of tuple-based methods to missing data** These tuple-based methods are defined and described assuming the input (e.g., gene trees or sequence alignments) has no missing data, and the statistics or the algorithm may not be fully defined if not all taxa are present. For example, for a given gene tree, the topology for quartet *ijkl* does not exist if one or more of the species is missing from the gene. Intuitively, if the method would have called for the calculation of a statistic on a particular set of taxa for a particular gene, it is not possible to calculate this if any taxon in the set is not present, so that gene should be excluded for purposes of that statistic. Thus, the natural extension of a tuple-based method (*F*,*α*) to inputs with missing data (i.e., when species are missing from genes) is as follows:

#### **Definition 1**

Let *A*=(*F*,*α*) be an *ℓ*-tuple species tree estimation method. The **natural extension** of *A* computes the summary statistics for a given set *B* of *ℓ* species based only on those genes that contain all the species in *B*.

**Type 1 and Type 2 *ℓ*-tuple methods** Since the set of summary statistics includes a real number for every tree *t* on *ℓ* species, we will let *F*_*t*_(*I*) denote the summary statistic computed by the function *F* for tree *t* given input *I*. For a set *B* of *ℓ* species drawn from the full set $\mathcal {X}$ of species, let *I*|_*B*_ denote the input set *I* restricted to *B*; thus, all species in $\mathcal {X} \setminus B$ are deleted entirely from the input. Then tuple-based methods can be characterized further depending on how they behave on such inputs. Specifically, we will partition *ℓ*-tuple methods (*F*,*α*) into two categories:

- Type 1: For all inputs *I*, all sets *B* of *ℓ* species from $\mathcal {X}$, and all trees *q* on *B*, $F_{q}(I)=F_{q}\left (I|_{B}\right)$.
- Type 2: There is at least one input *I*, one set *B* of *ℓ* species, and one tree *q* on *B* such that $F_{q}(I) \neq F_{q}\left (I|_{B}\right)$.

Thus, a Type 1 *ℓ*-tuple method has the property that deleting taxa from outside a set *B* does not impact the summary statistics it computes for any tree on *B*. Note that taxon deletion impacts both Type 1 and Type 2 methods, in that if enough taxa are deleted from enough genes then accuracy must decrease. As we will see, Type 1 methods are easier to analyze than Type 2 methods, and in particular it is easy to prove that a Type 1 method remains statistically consistent in the presence of missing data for some models of random taxon deletion. Most coalescent-based species tree estimation methods are Type 1 tuple-based methods; for example, ASTRAL, GLASS, METAL, MP-EST, STEAC, and SVDquartets are all Type 1 tuple-based methods. ASTRID, NJst, and STAR are Type 2 methods.

**Taxon-Deletion Models** Let $\mathcal {T}$ be a species tree on a set $\mathcal {X}$ of *n* species, with $\mathcal {X}=\left \{x_{i}\right \}_{i=1}^{n}$, and let *m* gene trees evolve within $\mathcal {T}$ under the multi-species coalescent model. We denote the set of gene trees by $\mathbf {T}=\left \{T_{i}\right \}_{i=1}^{m}$ and the set of genes by $\mathcal {G}=\left \{g_{i}\right \}_{i=1}^{m}$. To model taxon deletion, we let *g*_*i*_ denote an arbitrary gene and $Y_{i}=\left [Y_{i1},\dotsc, Y_{in}\right ]$ denote a random *n*-dimensional vector where

1 $$ Y_{ij}=\mathbb{I}_{\left\{ x_{j}\,\,\text{is\,\, present\,\, in}\,g_{i}\right\}}  $$

Here each individual *Y*_*ij*_ is a binary random variable that represents whether a species *x*_*j*_ is present for a random gene *g*_*i*_.

**Exchangeability** For the following lemma, we will assume that if gene tree *T*_*i*_ is generated before taxon deletion model *Y*_*i*_, then the post-deletion tree $T_{i}^{*}=T_{i} | Y_{i}$ is obtained by taking the subtree of *T*_*i*_ restricted to the set of leaves $\left \{x_{j} | Y_{ij}=1\right \}$ (i.e., the set of leaves corresponding to taxa that have not been marked for deletion by *Y*_*i*_). If *Y*_*i*_ is generated before *T*_*i*_, then $T_{i}^{*}$ is obtained by taking the same subtree $\mathcal {T}|Y_{i}$ of the species tree $\mathcal {T}$, and simulating a gene tree within this species subtree under the multi-species coalescent.

#### **Lemma 2**

If *T*_*i*_ and *Y*_*i*_ are independent, the two variables are exchangeable and the distribution of $T_{i}^{*}$ does not depend on the order of events.

#### *Proof*

If *Y*_*i*_ is generated first, then the conditional distribution of $T_{i}^{*}$ is equal to the distribution of gene trees under the multi-species coalescent on $\mathcal {T}|Y_{i}$, by definition. If *T*_*i*_ is generated first, then the pruning operations described above mean that $T_{i}^{*}$ will lie entirely within the subtree $\mathcal {T}|Y_{i}$. It remains to show that the probability of any given pattern of coalescence on the remaining branches is identical to the MSC under $\mathcal {T}|Y_{i}$. This follows from the memoryless property of coalescence under the MSC model: the probability of any two lineages originating within $\mathcal {T}|Y_{i}$ coalescing at any given point is not dependent on either lineage’s coalescent history. □

It should be noted by this model description, taxa are absent or present independently of the generation of the gene data, including tree topology and sequence evolution, and the two processes are exchangeable. Also, as is the case with the general multi-species coalescent model, gene trees evolve under a process that is *i.i.d.* with respect to one another. We will now define the three models for taxon deletion that were briefly described earlier: the *M*_*iid*_, the *M*_*fsc*_, and the *M*_*clade*_ models.

**The *i.i.d.* Model (*M***_***iid***_**)** *M*_*iid*_ is a family of models parameterized by *p*, with 0<*p*<1, where *p* is the probability that a random gene is present in a random species. For *M*_*iid*_ for parameter *p*, we assume that *Y*_*ij*_∼*Bernoulli*(*p*) for all genes *i* and all taxa *j*, and that *Y*_*ij*_ and *Y*_*kj*_ are independent for *k*≠*i*.

**The Full Subset Coverage Model (*M***_***fsc***_**)** *M*_*fsc*_ is a family of models parameterized by an integer *k*≥2. We assume that the taxon deletion process is *i.i.d.* across genes and furthermore that it is independent of the gene tree random variable, but we do not assume that it is *i.i.d.* across species. An *M*_*fsc*_ model for parameter *k* satisfies the property that for any subset *B* of at most *k* species there is a strictly positive probability *p*_*B*_ (that can depend on *B*) so that given a random gene, every member of *B* is present in the data for that gene with probability *p*_*B*_. Since the number of taxon sets of size at most *k* is finite, $p^{*} = \min \left \{p_{B}: B \subseteq \mathcal {X}, |B| \leq k\right \} > 0$; hence, every taxon subset of size at most *k* appears in a random gene with probability at least *p*^∗^. Note that every *M*_*iid*_ model satisfies the property of being an *M*_*fsc*_ model for every *k*.

**The Clade-Based Model (*M***_***clade***_**)** Every clade *c* in the species tree (including the clade containing all the species) has a strictly positive probability *p*_*c*_ of being selected, so that $\sum _{c} p_{c} = 1$. Then, for each gene, a clade is selected (under the above model), and all the species outside of the clade are deleted. Because clade selection is done on the species tree, the presence/absence of taxa is independent of the gene tree topology, and every subset has a strictly positive probability of appearing as a subset of the leafset of a random gene. Hence, the *M*_*clade*_ model of missing data falls under the *M*_*fsc*_ model. This model is based on pure gene birth model, where a gene is born on a branch in the species tree and then does not die; hence, the gene does not exist for taxa outside of the clade below the edge on which the gene was born, but appears at every leaf in the clade.

**Comparison to previous models of missing data** Several prior studies of the impact of missing data on phylogenomic analysis have been performed under the *M*_*iid*_ model; this model is referred to as **R** in [23] and as the “random allocation” model in [25]. Xi et al. [23] considered the **G** model, where missing data are concentrated in a subset of randomly chosen genes, and then taxa are deleted under an *i.i.d.* process from these genes. Xi et al. [23] also studied the **S** model, where missing data are allowed only in a subset of randomly chosen ingroup species, and that the genes are deleted from the selected species under an *i.i.d.* process. Note that the **S** and **G** models are *M*_*fsc*_ models. Other *M*_*fsc*_ models have been studied in [7, 24]. The *M*_*clade*_ model has been studied in [39] and used to explore supertree methods when there is no gene tree heterogeneity [36, 40–42].

**Results under an adversary model** We provide results under an adversary model of taxon deletion, showing that statistical consistency cannot be in general guaranteed under arbitrary models of missing data. We then provide results under the *M*_*iid*_ and *M*_*fsc*_ models of taxon deletion.

#### **Theorem 3**

Let taxon deletion be dependent on gene tree topology. There exists a dependency structure under which no method is statistically consistent.

#### *Proof*

Let $\mathcal {T}$ and $\mathcal {T}'$ be two species trees with different unrooted tree topologies; note that the unrooted topology for $\mathcal {T}'$ appears with strictly positive probability under the MSC for species tree $\mathcal {T}$. For each gene *g*_*i*_ (with true gene tree topology *t*_*i*_), consider the dependency structure where all taxa are present in the data for *g*_*i*_ with probability 1 if the topology of *t*_*i*_ is identical to $\mathcal {T}'$, and all taxa are absent with probability 1 otherwise. Thus, data for gene *g*_*i*_ is observed if and only if *t*_*i*_ has the same topology as $\mathcal {T}'$; in other words, the model species tree topology is not identifiable under this dependency structure. Hence, the distribution of observed gene data is not unique to the model species tree.

The identifiability condition (that the sampling distribution of observed data be uniquely determined by the model being estimated) is a natural precondition of any proof of statistical consistency for any method. That is, if two distinct parameter values yield identical data, an algorithm can return only one and will thus by necessity fail on some part of the parameter space. In particular, we have shown that two different model species trees that have different topologies define the same distribution of observed data (e.g., gene tree topologies) under this dependency structure. Therefore, it is not possible to have a statistically consistent method for species tree topology estimation under this dependency structure. □

Therefore, Theorem 3 demonstrates that a dependence between gene tree topology and taxon presence can quickly unravel statistical consistency guarantees in the absence of additional assumptions.

**Results for Type 1 methods under *M***_***fsc***_ We now discuss the statistical consistency guarantees of Type 1 tuple-based methods. As we will see, most of the tuple-based methods remain statistically consistent even in the presence of missing data, as long as the process that generates the missing taxa is well behaved (e.g., not generated by an adversary that biases the method towards the wrong tree).

Let *A*=(*F*,*α*) be a Type 1 *ℓ*-tuple method that satisfies the following properties:

- (i) For all model species trees $\mathcal {T} = (T,\Theta)$, as the number *m* of genes increases, $F(I_{m}) \overset {p} \longrightarrow F_{0}$, where *F*_0_ is a constant vector parameterized by $\mathcal {T}$.
- (ii) There exists *δ*>0 such that for all vectors of summary statistics *F*_1_ satisfying $L_{\infty }\left (F_{1},F_{0}\right) < \delta $, *α*(*F*_1_)=*α*(*F*_0_)=*T*.

#### **Theorem 4**

Let *A*=(*F*,*α*) be a Type 1 *ℓ*-tuple species tree estimation method satisfying the two properties (i) and (ii) above, and assume that the number of species is at least *ℓ*. The natural extension of *A* is statistically consistent under *M*_*fsc*_ with parameter *k*≥*ℓ*, and thus also under *M*_*iid*_ for any parameter *p*.

#### *Proof*

Let $\mathcal {T}=(T,\Theta)$ be the model species tree, *I*_*m*_ be the input dataset containing *m* genes, and *C* be the number of summary statistics computed by algorithm *A*=(*F*,*α*) on input *I*_*m*_. Since *A*=(*F*,*α*) satisfies condition (i) when there are no missing data, then as the number of genes *m* increases, $F(I_{m}) \overset {p} \longrightarrow F_{0}$, where *F*_0_ is a vector of constants. We will denote the *i*^*th*^ summary statistic computed on input *I*_*m*_ by *F*_*i*_(*I*_*m*_) and the *i*^*th*^ component of *F*_0_ by $F_{0_{i}}$. We write $F(I_{m})=\left (F_{1}\left (I_{m}|_{\mathbf {x}_{1}}\right),\dots,F_{C}\left (I_{m}|_{\mathbf {x}_{C}}\right)\right)$, where **x**_*i*_ denotes a particular set of *ℓ* taxa. In other words, since *A* satisfies condition (i) when there are no missing data, for all $i=1,\dots,C$ there exist a constant $F_{0_{i}}$ such that $F_{i}(I_{m}|_{\mathbf {x}_{i}})\overset {p}{\longrightarrow }F_{0_{i}}$ as $m \rightarrow \infty $. Since the data for each gene are independent of all others, to prove statistical consistency under the *M*_*fsc*_ model we merely require that $I_{m}|_{\mathbf {x}_{i}}\phantom {\dot {i}\!}$ include an infinite number of genes as $m\rightarrow \infty $. Under the *M*_*fsc*_ model, $Pr\left [\mathbf {x}_{i} \subseteq L(g)\right ] >0$ for every gene *g* (where $\mathcal {L}(g)$ denotes the set of species for gene *g*). Hence, by the Borel-Cantelli lemma, the number of genes that include all *ℓ* taxa in **x**_*i*_ will also approach infinity. Thus $I_{m}|_{\mathbf {x}_{i}}\phantom {\dot {i}\!}$ will include an infinite number of genes, and $F\left (I_{m}|_{\mathbf {x}_{i}}\right)\overset {p}{\longrightarrow }F_{0_{i}}$. By the definition of the natural extension of *A*, *α* does not change under deleted taxa. Since *A* satisfies condition (ii), ∃*δ*>0 such that ∀*F*_1_ with $L_{\infty }\left (F_{1},F_{0}\right) < \delta $, *α*(*F*_1_)=*T*, and so the natural extension of *A* is statistically consistent under *M*_*fsc*_. Since *M*_*iid*_ is a subset of *M*_*fsc*_, it is also statistically consistent under *M*_*iid*_. □

#### **Corollary 5**

ASTRAL, METAL, MP-EST, and STEM are statistically consistent under the MSC even when taxa are deleted under an *M*_*fsc*_ model, provided that each is run in exact mode (i.e., they find optimal solutions to their respective optimization criteria) and that the conditions necessary for statistical consistency when there are no missing data are also present.

SVDquartets [43] is a popular species tree estimation method that operates by computing quartet trees, and then combines the quartet trees using a quartet amalgamation method. However, it is not yet established that the quartet tree estimation method used in SVDquartets is statistically consistent under the MSC, even when there are no missing data, and so the statistical consistency of SVDquartets under the MSC is still an open problem. Although it is likely that the quartet tree estimation method used by SVDquartets is statistically consistent under the MSC, since there is no proof yet of this assertion, we cannot infer anything about the statistical consistency of SVDquartets under more general conditions.

**Statistical consistency of versions of ASTRAL under *M***_***fsc***_ ASTRAL-I [5] and its improved version, ASTRAL-II [6] are coalescent-based methods for estimating species trees that take unrooted gene trees as input, and return a tree that minimizes the quartet tree distance to the input gene trees. Each can be run in either exact mode or in a heuristic setting. In the exact mode, each is guaranteed to find an optimal tree – i.e., one that minimizes the quartet tree distance to the input set of unrooted gene trees –and is established to be statistically consistent under the MSC when there are no missing data. The heuristic settings operate by defining a set of “allowed bipartitions” (*β* in this paper), which means that the search space can only consider species trees that draw all their bipartitions from *β*. The heuristic settings are also statistically consistent under the MSC when there are no missing data, provided that the set *β* contains all the bipartitions from the input gene trees (which is guaranteed for both ASTRAL-I and ASTRAL-II).

The important difference between ASTRAL-I and ASTRAL-II is how the set *β* is defined, and ASTRAL-II explicitly enlarges the set *β* compared to ASTRAL-I when the input gene trees can be incomplete (i.e., when some species are missing from some gene trees). Because the search space is constrained using the input gene trees, the two ASTRAL algorithms depends on the input in a way that makes the analysis of their statistical guarantees non-trivial.

We will show that although ASTRAL-I and ASTRAL-II are statistically consistent under the *M*_*iid*_ model, there are some *M*_*fsc*_ models for which neither is statistically consistent. We then present some modifications to ASTRAL-I that differ from ASTRAL-I only in how the set *β* is defined, and show that these modifications are statistically consistent under many (but not all) *M*_*fsc*_ models.

**Notation** We let $\mathcal {X}$ denote the full set of species and $\mathcal {X}'$ denote an arbitrary subset of $\mathcal {X}$. Every tree *t* we consider is assumed to be a binary unrooted tree with leaves taken from a subset of $\mathcal {X}$, and as earlier we denote the leafset of *t* by $\mathcal {L}(t)$. Each edge of *t* defines a **bipartition** of the set $\mathcal {L}(t)$ (denoted by *B*|*B*^′^, for some set $B \subseteq \mathcal {X}$ and $B' = \mathcal {L}(t) \setminus B$) obtained by deleting the edge but not its endpoints from *t*. We will refer to the set of all these bipartitions as *Bip*(*t*), and the set of halves of the bipartitions of *t* as the **clades** of *t*. (Note that the term “clades” is normally used only in the context of rooted trees, but we extend the term here to allow us to refer to halves of bipartitions using the same term). We let $T_{\mathcal {X}}(\beta)$ denote the set of unrooted binary trees on leafset $\mathcal {X}$ that satisfy $Bip(t) \subseteq \beta $. If *β* is not provided, then we assume the set of unrooted binary trees is not constrained and we let $T_{\mathcal {X}}$ denote the set of all unrooted binary trees on leafset $\mathcal {X}$.

We let *Q*(*t*) denote all 4-leaf homeomorphic subtrees of *t* induced by a set of four leaves in *t*. Since we assume all trees *t* are binary in this study, it follows that *Q*(*t*) contains only binary quartet trees. Let $\mathcal {Q}$ be the set of all ${{n}\choose {4}}$ 4-taxon subsets of the taxon set $\mathcal {X}$. Let $q\in \mathcal {Q}$, let *t* be an arbitrary (binary) tree topology on $\mathcal {X}$, and let *Top*(*q*,*t*) denote the induced homeomorphic quartet tree topology for quartet *q* in *t*.

#### **Definition 6**

**ASTRAL Optimization Problem**

**Input**: Taxon set $\mathcal {X}=\{x_{i}\}_{i=1}^{n}$, unrooted binary gene trees $t_{1},\dotsc,t_{m}$, and set *β* of allowed bipartitions of $\mathcal {X}$

**Output**: Unrooted binary tree *T* where

$$ T=\arg\max_{t\in T_{\mathcal{X}}(\beta)} \sum_{q\in\mathcal{Q}}\sum_{i=1}^{m}\mathbb{I}_{\left\{Top(q,t)=Top\left(q,t_{i}\right)\right\}} $$

ASTRAL-I and ASTRAL-II differ in how they define the default set *X* of allowed bipartitions and they also use slightly different dynamic programming techniques to assemble the optimal tree from the bottom up. To run ASTRAL-I or ASTRAL-II in exact mode, the set *β* is defined to be all bipartitions on $\mathcal {X}$. In the default version of ASTRAL-I (referred to as the “heuristic version”), *β* is the set of all bipartitions that appear in any gene tree. Hence, when there are no missing data, then as the number of genes increases, the set *β* will include all possible bipartitions on the taxon set with probability converging to 1 (and hence in particular the bipartitions in the true species tree). However, when there are missing data, then proving that the set *X* contains all the bipartitions in the species tree takes some care. In particular, if every gene tree is incomplete, then no bipartition in any gene tree is a bipartition of the full set of taxa, and so this default setting will not enable a statistically consistent estimation method.

**Modifications to ASTRAL** We will modify ASTRAL-I by changing how it defines the set *β* of allowed bipartitions. Each of these modifications simply uses the input set of gene trees to compute a set of additional bipartitions, which are then added to the default setting for *β* that is computed by ASTRAL-I. Hence, these extra bipartitions could also be added to ASTRAL-II.

Note that ASTRAL-I, ASTRAL-II, and hence also these modifications, when run in heuristic mode, are different from the species tree estimation methods described previously, in that *α* depends not only on the summary statistics *F*(*I*) but also on the input data *I*. Therefore, we denote the output of the function by *α*(*F*,*I*).

The first modification we present is the simplest: For every clade $C\subset \mathcal {X}$ occurring in a gene tree, we add the bipartition *C*|*C*^′^ where $C' = \mathcal {X}\setminus C$, to its set *β*. (Note that since the trees in this problem are unrooted, a clade and one half of a bipartition are equivalent concepts). This is a trivial extension of the algorithm for a model of incomplete genes and one that expands the conditions under which the method is consistent, as we will see below. We refer to this particular modification as ASTRAL _*mod*1_.

#### **Theorem 7**

(1) ASTRAL _*mod*1_, ASTRAL-I, and ASTRAL-II, all run in default heuristic mode, are statistically consistent under the MSC for any *M*_*iid*_ model of taxon deletion. (2) ASTRAL-I is not statistically consistent under an *M*_*fsc*_ model with parameter *k* if the number of species is greater than *k* and ASTRAL-I is run in default heuristic mode. (3) ASTRAL _*mod*1_ is statistically consistent under any *M*_*fsc*_ model of taxon deletion with parameter *k* if the number *n* of species is at most 2*k*.

#### *Proof*

(1) Let $\mathcal {T}$ be a model species tree on *n* species, and consider taxon deletion under some *M*_*iid*_ model. We will show that there is non-zero probability that every bipartition in the species tree appears in the set *β* (which defines the search space) computed by ASTRAL-I in its default setting. Since the search space computed by ASTRAL-I is a subset of the search space computed by ASTRAL-II and ASTRAL _*mod*1_, the result will follow. Recall that ASTRAL-I includes all bipartitions *C*|*C*^′^ in the set *β* that appear in any input gene tree. Under the MSC model, every bipartition appears in some gene tree with probability increasing to 1 as the number of genes increases. Under any *M*_*iid*_ model, for every subset of taxa, the probability that none of the taxa in the subset are deleted is strictly greater than 0. Hence, under *M*_*iid*_, the set *β* of bipartitions allowed in the ASTRAL-I search space will converge to the set of all possible bipartitions. Therefore, ASTRAL-I is statistically consistent under *M*_*iid*_. Since the sets of bipartitions computed by ASTRAL _*mod*1_ and ASTRAL-II contain the set of bipartitions computed by ASTRAL-I, it follows that ASTRAL-II and ASTRAL _*mod*1_ are also statistically consistent under *M*_*iid*_.

(2) Now consider the *M*_*fsc*_ model with parameter *k*. Let *n*>*k*, and let the taxon deletion process be such that every gene has exactly *k* taxa (e.g., *k* taxa sampled uniformly, a valid model under *M*_*fsc*_). When ASTRAL-I is run in heuristic mode, it will compute a set *β* that contains no bipartitions on the set $\mathcal {X}$ of species, and so cannot be statistically consistent.

(3) We show that ASTRAL _*mod*1_ run in heuristic mode is statistically consistent under any *M*_*fsc*_ model with parameter *k* when *n*≤2*k*. Let *C*|*C*^′^ be an arbitrary bipartition on $\mathcal {X}$, and assume without loss of generality that |*C*|≤*k*. Hence, under *M*_*iid*_, the probability that all the taxa in *C* appear in a random gene tree is strictly positive. Under the MSC, any bipartition on $\mathcal {X}$ appears in a random true gene tree with strictly positive probability. Since this process is independent from the removal of taxa, and since there is non-zero probability that all members of a clade appear in the gene tree, the probability is non-zero that the set *C* appears as a clade in a random gene tree.

Hence, as the number *m* of gene trees increases, the probability approaches 1 that *C* appears as a clade in at least one gene tree. Thus the probability approaches 1 that the set *β* computed by ASTRAL _*mod*1_ will contain *C*|*C*^′^, where $C' = \mathcal {X} \setminus C$. Therefore, ASTRAL _*mod*1_, run in heuristic mode, will be statistically consistent under the *M*_*fsc*_ model with parameter *k*, provided that the number *n* of species is at most 2*k*. □

#### **Theorem 8**

ASTRAL-I and ASTRAL _*mod*1_, when run in heuristic mode, are not statistically consistent under the *M*_*fsc*_ class of models with parameter *k*, if the number of species *n*>2*k*.

#### *Proof*

Consider a model of taxon deletion where every gene tree has exactly *k* taxa, selected at random from the full set of taxa. This model satisfies the conditions of the *M*_*fsc*_ models with parameter *k*. Now assume *k*<⌊*n*/2⌋.

Let $\mathcal {T}$ be a caterpillar tree on a set $\mathcal {X}$ of *n* taxa. Then $\mathcal {T}$ contains a clade *B* of size ⌊*n*/2⌋ whose complement is at least as large; hence both *B* and $\mathcal {X} \setminus B$ have more than *k* species. Hence, under this model of taxon deletion, neither *B* nor $\mathcal {X} \setminus B$ will be in any gene tree. Hence, the bipartition *B*|*B*^′^ (where $B'=\mathcal {X} \setminus B$) will not be in *β* (the constraint on the search space) as computed by ASTRAL-I and ASTRAL _*mod*1_. Hence, neither ASTRAL-I nor ASTRAL _*mod*1_ can recover the true species tree under this random taxon deletion model. □

Although ASTRAL _*mod*1_ is not statistically consistent under some *M*_*fsc*_ models, it is possible that other modifications can be statistically consistent under *M*_*fsc*_ models. In particular, suppose *Φ* is a species tree method that is statistically consistent under the *M*_*fsc*_ model. Then the simple approach of using *Φ* to estimate the species tree and then adding its bipartitions to the set *β* suffices to make ASTRAL consistent under the *M*_*fsc*_ model. We refer to this as the ASTRAL+ *Φ* method, and summarize this observation in the theorem below.

#### **Theorem 9**

Let *Φ* be a species tree estimation method that is statistically consistent under the MSC when taxa are deleted under the *M*_*fsc*_ model. Then ASTRAL+ *Φ*, the modification of ASTRAL-I obtained by adding the bipartitions of the species tree computed by *Φ* to the set *β*, is statistically consistent under the MSC when taxa are deleted under the *M*_*fsc*_ model.

**Statistical consistency of ASTRID and NJst under *M***_***iid***_ As noted earlier, ASTRID, NJst, and STAR are Type 2 methods, and the proofs we provided of statistical consistency for Type 1 tuple-based methods do not apply to these methods (or other Type 2 methods). We will show ASTRID and NJst remain statistically consistent under the *M*_*iid*_ models of taxon deletion; however, the statistical consistency of these methods under more general *M*_*fsc*_ models is unknown.

NJst and ASTRID are distance-based methods that use the average topological “internode” distance between taxa in the gene trees, where the internode distance between two taxa *x*_*i*_ and *x*_*j*_ in a tree (denoted *ρ*(*x*_*i*_,*x*_*j*_)) is the number of individual nodes along the path from the leaves corresponding to *x*_*i*_ and *x*_*j*_. NJst and ASTRID are not Type 1 methods, because the internode distance for two taxa *x*_*i*_ and *x*_*j*_ can be affected by the presence or absence of a third taxon.

NJst and ASTRID are formally 2-tuple methods, and each uses an algorithm to compute a tree given ${{n}\choose {2}}$ pairwise distances (collectively, the “distance matrix”). We now state some well-known properties of distance methods for reference in the proof below. For a tree with topology *T*=(*V*,*E*) on *n* taxa and edge weights *l*_*e*_, *e*∈*E*, if the distance for any two taxa *j* and *k* is equal to the sum of the edge weights over edges in the shortest path between leaves *j* and *k*, then neighbor joining will return a tree with topology *T*, and the distance matrix is said to be **additive** on the topology *T*. An equivalent definition of an additive matrix is as follows:

#### **Definition 10**

**Additivity and The Four Point Condition** A square matrix *D*=[*d*_*ij*_] is said to be **additive** if there is a tree *T* with *n* leaves and non-negative branch lengths so that *d*_*ij*_ is the total weighted path length between leaves *i* and *j*. When such a tree *T* exists, we also say that *D* is additive on the topology *T*. The **Four Point Condition** is said to hold for a square matrix *D* if and only if for all sets of four indices $\left \{i,j,k,l\right \}$, the median and maximum of the three pairwise sums *d*_*ij*_+*d*_*kl*_,*d*_*ik*_+*d*_*jl*_,*d*_*il*_+*d*_*jk*_ are equal.

It is also known that a matrix is additive if and only if it satisfies the Four Point Condition [44]. Furthermore, if *D* is additive and corresponds to an edge-weighting of tree *T*, then for a given set of four indices {*i*,*j*,*k*,*l*}, *d*_*ij*_+*d*_*kl*_<*d*_*ik*_+*d*_*jl*_=*d*_*il*_+*d*_*jk*_ if and only if the quartet tree induced by *T* on these four indices has an edge separating the leaves for *i*,*j* from the leaves for *k*,*l*.

Distance-based tree estimation methods operate by computing trees from matrices of estimated distances. If the “distance matrix” is additive, most distance-based methods are guaranteed to return a tree topology that can realize the additive matrix. Furthermore, if instead of being given the additive matrix *D* as above we are given $\hat {D}=d_{ij}+\varepsilon _{ij}$, where *ε*_*ij*_ is an unknown noise term such that for all $i,j\in \mathcal {S}\}$, $|\varepsilon _{ij}|<\frac {1}{2} \min \{ d_{ab}: a,b\in \mathcal {S}\}$, then neighbor joining applied to the matrix $\hat {D}$ will also return the topology *T* with probability 1 [45]. ASTRID is similar to NJst in that it computes the same internode distance matrix but then uses FastME to compute the species tree; since FastME also has some error tolerance [46], ASTRID is also guaranteed to return the species tree when the internode distance matrix is close enough to the matrix *D*. Therefore, to prove statistical consistency for both ASTRID and NJst, it suffices to show that the average internode distance matrix computed from a set of input gene trees converges to an additive matrix on the true topology as the number of genes increases. This assertion has already been established when there are no missing data [47, 48], but not when taxa are deleted from the gene trees.

#### **Theorem 11**

Assume that taxa are absent from the data for each gene according to the *M*_*iid*_ model. Then NJst and ASTRID are statistically consistent under the MSC.

We begin with a lemma that will be helpful for establishing the proof. Note that since $\rho \left (x_{i},x_{j}\right)$ is undefined when either of *x*_*i*_ or *x*_*j*_ is removed, the expectation of $\rho \left (x_{i},x_{j}\right)$ is formally undefined as long as the probability of either being deleted is nonzero. We nonetheless use the notation $\mathbf {E}\left [\rho (x_{i},x_{j})\right ]$ in the lemma and proofs below, which will refer implicitly to the conditional expectation on the event that neither *x*_*i*_ nor *x*_*j*_ is removed.

#### **Lemma 12**

Under the MSC and *M*_*iid*_, let *a*, *b*, *c* and *d* be four taxa. Consider the event in the coalescent probability space, denoted as $\mathcal {E}_{abcd}$, in which the lineages of these taxa have entered a common population and no pair have coalesced with one another. Denote the points on each respective lineage in which they enter the common population as *A*, *B*, *C*, and *D*. Let *Y* be the random variable representing the taxon deletion process. Then for any two taxa $\{i,j\} \subset \{a,b,c,d\}$ and respectively $\left \{ I,J\right \} \subset \{A,B,C,D\}$:

$$\begin{aligned} \mathbf{E}\left[ \rho(i,j) | \mathcal{E}_{abcd} \right] &=\mathbf{E}_{Y}\left[\rho(i,I)|\mathcal{E}_{abcd}\right]\\ &\quad + \mathbf{E}_{Y}\left[\rho(j,J)|\mathcal{E}_{abcd}\right] + K \end{aligned} $$

where *K* is a constant that does not depend on the identities of *i* and *j*.

#### *Proof*

Consider any rooted gene tree *g* meeting the condition of event $\mathcal {E}$, and let *G* be the subtree of *g* sitting between the points *A*, *B*, *C* and *D* and the root. Equivalently, *G* is obtained by deleting the subtrees of *g* below points *A*,*B*,*C*, and *D*. The topology of *G* in this model, given that $g\in \mathcal {E}$, is determined by a set of *i.i.d.* exponentially distributed random variables corresponding to the pairwise times-to-coalescence of all remaining lineages concurrent with and including {*A*,*B*,*C*,*D*}. By De Finetti’s theorem, the probability density function of *G* is unique up to a permutation of the indices of the random variables. Thus for any $\{I,J\}\subset \{A,B,C,D\}$, since *ρ*(*I*,*J*) is dependent only on the topology of *G*, the probability density of *ρ*(*I*,*J*) does not depend on the identity of *I* and *J* so that $\mathbf {E}\left [ \rho (I,J)|\mathcal {E} \right ]$ does not depend on *I* or *J*, and we let the common value for these expectations be denoted by *K*. □

#### *Proof of Theorem 11*

Recall that ASTRID and NJst compute a matrix of average internode distances (defined by the summary statistics *ρ*(*x*_*i*_,*x*_*j*_)), and then NJst uses neighbor joining and ASTRID uses FastME to compute a tree. Since both neighbor joining and FastME return the tree topology *T* when given a matrix that is sufficiently close to an additive matrix corresponding to an edge-weighting of tree *T*, to prove that these methods are statistically consistent under the *M*_*iid*_ missing data model, it suffices to establish the following:

1. For a gene tree generated under the coalescent process in the MSC followed by the removal of taxa subject to *M*_*iid*_, the expected value of the summary statistics *ρ*(*x*_*i*_,*x*_*j*_) for each pair of taxa *x*_*i*_,*x*_*j*_ form an additive matrix that defines the topology of the species tree.
2. The statistics themselves converge to their expectations as the number *m* of genes approaches infinity.

The second property follows from the *i.i.d.* generation of gene trees and the weak law of large numbers; hence, we need only establish the first property.

Let *G* be a random gene tree on *n* taxa generated by the MSC on species tree $\mathcal {T}$, and let *Y*_*n*_∼*M*_*iid*_ with probability of deletion *p*. We will show that for an arbitrary set of four taxa {*x*_1_,*x*_2_,*x*_*a*_,*x*_*b*_}, the expectations over *G* and *Y*_*n*_ of the internode distances are additive on the species tree, which we check by confirming that they obey the Four Point Condition for the species tree $\mathcal {T}$. In what follows, we will refer to this by saying that the expectations “obey the Four Point Condition”, with the understanding that this is with reference to the species tree $\mathcal {T}$. Importantly, the event $\mathcal {E}$ defined in Lemma 12 includes all cases in which the homeomorphic quartet subtree topology in the gene tree does not match that of the species tree, and implies that the Four Point Condition holds in these cases. As a result, it suffices to show that the Four Point Condition holds when the homeomorphic quartet subtree in *G* is identical to the topology in the species tree, which we assume without loss of generality has topology *x*_1_*x*_2_|*x*_*a*_*x*_*b*_ (i.e., the species tree and the gene tree *G* both have edges that separate *x*_1_,*x*_2_ from *x*_*a*_,*x*_*b*_). We will show this by induction on the number *n* of taxa, and begin with the smallest non-trivial case, *n*=4.

**Base Step:** **n=4**. For *n*=4 we can write the expected values of the internode distances in closed form and check the Four Point Condition directly:

$$\begin{array}{*{20}l} \begin{array}{ll} {}\mathbf{E} \left[\rho(x_{1},x_{2})\right]&=2-p^{2}\\ {}\mathbf{E} \left[\rho(x_{a},x_{b})\right]&=2-p^{2}\\ {}\mathbf{E} \left[\rho(x_{1},x_{a})\right]&=3(1-p) (1-p) + 2\left(p + p - p^{2}\right) + 1\left(p^{2}\right)\\ &=3-3p-3p +3p^{2}+ 2p +2p - 2p^{2} + p^{2}\\ &=3-2p +2p^{2}\\ {}\mathbf{E} \left[\rho(x_{2},x_{b})\right]&=3-2p +2p^{2} \end{array} \end{array} $$

Thus, to test that the Four Point Condition holds, we have:

$$\begin{array}{*{20}l} \begin{array}{ll} \mathbf{E} \left[\rho(x_{1},x_{2})\right] + \mathbf{E} \left[\rho(x_{a},x_{b})\right] &= 4 - p^{2} - p^{2}\\ &=4-2p^{2}\\ \mathbf{E} \left[\rho(x_{1},x_{a})\right]+\mathbf{E} \left[\rho(x_{2},x_{b})\right]&=6-4p +4(p^{2})\\ \mathbf{E} \left[\rho(x_{2},x_{a})\right]+\mathbf{E} \left[\rho(x_{1},x_{b})\right]&=6-4p +4(p^{2})\\ \end{array} \end{array} $$

Thus the Four Point Condition holds for *n*=4.

**Induction Step:** Assume that for a set $S_{n} = \left \{x_{1}, x_{2}, \ldots, x_{n}\right \}$ of *n* taxa, the expected value of the matrix *D*=[*ρ*(*x*_*i*_,*x*_*j*_)] for a random gene tree *G*_*n*_ and taxon-removal variable *Y*_*n*_ is additive on the true species tree. That is, for any set of *n* taxa under the MSC and *M*_*iid*_ and any quartet {*a*,*b*,*c*,*d*}, the Four Point Condition holds for $\mathbf {E}_{n}\left [\rho (i,j)\right ]$, *i*,*j*∈{*a*,*b*,*c*,*d*}, a fact that we will use below (here **E**_*n*_ denotes the expectation operator under *n* taxa for notational clarity). We will now show that the same holds for a set of size *n*+1.

Let *x*_*n*+1_ be a new taxon and let *G* be generated under the MSC on $S_{n}\cup \left \{x_{n+1}\right \}$ with taxon-removal variable *Y*_*n*+1_=(*Y*_*n*_,*y*_*n*+1_) where *y*_*n*+1_∼*Bernoulli*(*p*) in accordance with *M*_*iid*_. Our approach will be to define three cases, each of which have non-zero probability, and show that regardless of the placement of *x*_*n*+1_ in the species tree, the Four Point Condition holds on $\left \{x_{1},x_{2},x_{a},x_{b}\right \}$.

*Case 1:* *x*_*n*+1_ is deleted from *G* (i.e. *y*_*n*+1_=1). In this case the conditional values of $\mathbf {E}_{n+1}\left [\rho (x,x')\right ]$ for *x*≠*x*^′^, $x,x'\in \left \{x_{1},x_{2},x_{a},x_{b}\right \}$ are identical to the case with *n* taxa, and thus by our assumption they obey the Four Point Condition.

*Case 2:* *x*_*n*+1_ is not deleted and it coalesces (with some other lineage) on a branch that is *not* on the homeomorphic quartet subtree of *G* for the quartet $\left \{x_{1},x_{2},x_{a},x_{b}\right \}$. In this case, the coalescence event for *x*_*n*+1_ does not add to the internode distances along any of the branches connecting any two members of the quartet. As a result, again the conditional values of $\mathbf {E}\left [\rho (x,x')\right ]$ for *x*≠*x*^′^, $x,x'\in \left \{x_{i},x_{j},x_{k},x_{l}\right \}$ are identical to the case with *n* taxa, where again they obey the Four Point Condition, by the induction assumption.

*Case 3:* *x*_*n*+1_ is not deleted and coalesces directly with a branch on the induced quartet subtree of *G* for the quartet $\left \{x_{1},x_{2},x_{a},x_{b}\right \}$. This case is non-trivial and requires some analysis. For shorthand, we will refer to the homeomorphic quartet subtree of *G* restricted to $\left \{x_{1}, x_{2},x_{a},x_{b}\right \}$ as simply *q*. For a pair of taxa *i*,*j*∈*S*_*n*_ the value of the expected internode distance in the presence of *x*_*n*+1_ is:

2 $$\begin{array}{*{20}l} \mathbf{E}_{n+1}\left[\rho(i,j)\right]=\mathbf{E}_{n}\left[\rho(i,j)\right]+P(i,j) \end{array} $$

where *P*(*i*,*j*) denotes the probability that *x*_*n*+1_ coalesces on a branch in the path from *i* to *j*, which would cause *ρ*(*i*,*j*) to increase by 1. This quantity is non-trivial and depends jointly on both the topology of *Gand* the value of *Y*_*n*_. However, since the first term obeys the Four Point Condition by the induction hypothesis, the proof depends on showing that *P*(*i*,*j*) does as well.

To do that, we will first partition the joint probability space of the MSC and *M*_*iid*_, denoted as $\mathcal {G}$ and assign each part of this partition to one of the five branches in the subtree *q*, then show that for $\{i,j\} \subset \{x_{1},x_{2},x_{a},x_{b}\}$, *P*(*i*,*j*) can be expressed as the sum of probabilities assigned to the branches between *i* and *j*. We will label the branches as *b*_1_, *b*_2_, *b*_*a*_, and *b*_*b*_ for the four outer branches and *b*_*m*_ for the middle branch.

**Fig. 2:**
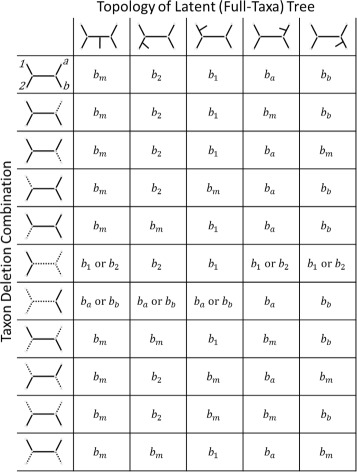
Table of attachment branches for Theorem 11. Table of attachment branches given the latent 5-taxon topology and set of deleted taxa from *q*, assuming no other taxa coalesce closer to the inner nodes, for Theorem 11. Branches (in rows) given by dotted lines are pruned as a result of taxon deletion. The notation *b*_*x*_ (for *x*=*a*,*b*,1,2) refers to the branch of the quartet tree on leaves *a*,*b*,1,2 incident with leaf *x*, and *b*_*m*_ refers to the middle branch of the quartet tree

Figure 2 describes the partition based on the branch with which *x*_*n*+1_ coalesces (in each column) and the outcome of the taxon-removal process (in each row). For the illustrations in the header of each row, a branch represented with a dotted line represents the case that **all** lineages coalescing along that branch other than *x*_*n*+1_ are fully removed by the taxon-removal process. The positions of the relative taxa are given in the illustration in the first row header, and all taxon-removal outcomes that allow at least one *ρ*(*i*,*j*), *i*,*j*∈{*x*_1_,*x*_2_,*x*_*a*_,*x*_*b*_} to be measured are represented in the rows. The assignment is not unique, as implied by the entries with offer two possibilities, but for the proof either will work so we consider the default to be the first branch listed.

For an event $(G,Y_{n+1}) \in \mathcal {G}$, denote the assignment of $\left (G,Y_{n+1}\right)$ to branch *b*_*e*_ as $(G,Y_{n+1}) \rightarrow b_{e}$, and for that branch let

$$p_{e} = P\left(\{(G,Y_{n+1})\in\mathcal{G} | (G,Y_{n+1}) \rightarrow b_{e}\} \right).$$

In this way, for any pair of taxa *i*,*j*∈{*x*_1_,*x*_2_,*x*_*a*_,*x*_*b*_}, the probability that *ρ*(*i*,*j*) is incremented by 1 in the presence of *x*_*n*+1_ is given precisely by the sum of *p*_*e*_ for set of edges *e* between *i* and *j* in the quartet subtree *q*. This should be apparent by visual inspection of the table. Thus, *P*(*i*,*j*) in (2) is the sum of positive edge weights on the topology *x*_1_*x*_2_|*x*_*a*_*x*_*b*_, which is also the species tree topology on these four taxa, and so is additive on the species tree. Hence, it meets the Four Point Condition, completing the proof. □

This proof was only provided under the *M*_*iid*_ model of taxon deletion because the independence of taxon deletion (between taxa as well as from gene tree generation) was used when we noted that

3 $$ \mathbf{E}_{n+1}\left[\rho(x,x')\right]=\mathbf{E}_{n}\left[\rho(x,x')\right]+p_{xx'}  $$

in case 3. Independence implies that the marginal probability distribution of *ρ*(*x*,*x*^′^) for *n* taxa is identical to the conditional distribution given the information that *x*_*n*+1_ has not been deleted.

While it is not strictly necessary for (3) to hold, counterexamples can be difficult to construct, and thus it is non-trivial to characterize conditions that may be weaker than pure independence and still imply that ASTRID and NJst are statistically consistent. It is an open question whether ASTRID and NJst are statistically consistent under any more general model (e.g., under the *M*_*fsc*_ model). It is also an open question whether other Type 2 methods (e.g., STAR) are statistically consistent under the *M*_*iid*_ model of taxon deletion.

### Results of the simulation study

The theoretical results above establish that various species tree estimation methods are statistically consistent under the MSC model in the presence of missing data under the *M*_*iid*_ model, and, in some cases, also under the *M*_*fsc*_ model. Statistical consistency is a statement about performance in the limit, as the amount of data increases, but does not directly address performance on finite data. The question we address in this simulation study is: “How quickly does species tree error decrease as the number of genes increases under models of missing data?”

We explored the performance of four species tree estimation methods under two speciation models: one where speciation was deep (toward the root) and the other where speciation was recent (toward the leaves). The results for datasets with deep speciation are shown here, and the results for datasets with recent speciation are shown in the Additional file 1. Nearly identical trends are observed for the two speciation rates; however, species trees estimated on datasets with recent speciation had somewhat reduced error rates, that is, recent speciation was a less challenging model condition.

We performed a sequence of experiments to evaluate the impact of missing data on species tree estimation. Our preliminary experiment evaluated the impact of missing data on the inputs given to species tree estimation methods (i.e., gene tree estimation error, discordance between true gene trees and the species tree, and the internode distance matrix computed by ASTRID). We then performed experiments evaluating the impact of taxon deletion on species tree estimation based on true gene trees (for the summary methods) and the true alignment (for SVDquartets). Our final experiment examines the impact of taxon deletion under both models when summary methods are given estimated gene trees rather than true gene trees.

**Fig. 3:**
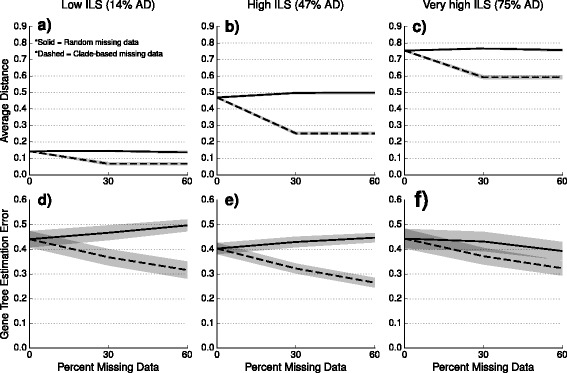
Impact of missing data on AD and GTEE values. Average distance (or AD, defined as the normalized RF distance between the true species tree and the true gene trees, averaged across all 1000 genes) and gene tree estimation error (or GTEE, defined as the normalized RF distance between the true and the estimated gene trees, average across all 1000 genes) are shown for increasing amounts of missing data in panels (**a**-**c**) and (**d**-**f**), respectively. Each column represents a different level of incomplete lineage sorting (ILS): panels (**a**) and (**d**) show datasets with low ILS, panels (**b**) and (**e**) show datasets with high ILS, and panels (**c**) and (**f**) show datasets with very high ILS. Lines represent the average over 20 replicate datasets, and filled regions indicate the standard error. Solid lines indicate the *M*_*iid*_ model of missing data, and dashed lines indicate the *M*_*clade*_ model of missing data. Note that datasets with 55% and 95% of genes with clade-based missing data had 34% and 59% total missing data, respectively. Datasets shown here have deep speciation events and 1000 genes; results for datasets with recent speciation are shown in Additional file 1

**Experiment 1: Evaluating the impact of taxon deletion on inputs to species tree estimation methods** We first evaluated the impact of taxon deletion on gene tree estimation error (GTEE), measured using the average normalized RF distance between true gene trees and estimated gene trees, and also on the ILS level, as measured using AD values; see Fig. 3. Deleting taxa under the *M*_*iid*_ model had only a small impact on GTEE and AD levels: on average, AD changed by at most 4% and GTEE changed by at most 7%. However, deleting taxa under the *M*_*clade*_ model resulted in a noticeable *decrease* for both GTEE and AD values: on average, AD decreased by as much as 22% (Fig. 3b, dashed line) and GTEE decreased by as much as 14% (Fig. 3e, dashed line).

We also explored the impact of taxon deletion on *D*^*A*^, the internode distance matrix ASTRID computes from the set of true gene trees given as input. Our theory establishes that as the number of genes increases, *D*^*A*^ converges to a matrix that is additive on the true species tree; however, the additive matrix it converges to depends on the model of taxon deletion. In particular, under the clade-based model of taxon deletion, the internode distance matrix converges to a specific additive matrix *D*^*T*^, defined by the true species tree with unit branch lengths; however, under the *M*_*iid*_ model the internode distance matrix converges to a different additive matrix (one that also fits the model species trees, but with different edge lengths). Therefore, the distance between *D*^*A*^ and *D*^*T*^ is a good proxy for the deviation from additivity for the internode distance matrix ASTRID calculates under the clade-based model, but a poorer (albeit potentially useful) proxy under the *M*_*iid*_ model. Furthermore, since ASTRID is guaranteed to return the true species tree if the input matrix is sufficiently close to *D*^*T*^, proximity to *D*^*T*^ may be predictive of topological accuracy for the ASTRID species tree.

Our experiment explored these issues for both models of missing data under three levels of ILS, three numbers of genes, and with varying amounts of missing data, but always for ASTRID applied to true gene trees. For each model condition, we computed a distance between *D*^*T*^ and *D*^*A*^ as follows:

$$\| E \|_{2} = \sqrt{\left|D^{T}_{R} - D^{A}_{R}\right|^{2}}, $$

where *M*_*R*_ denotes the restriction of matrix *M* to its upper right triangle.

**Fig. 4:**
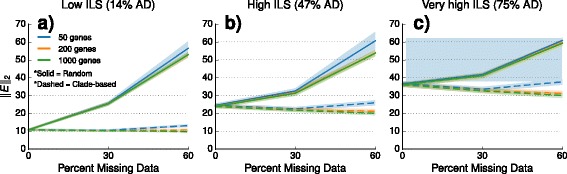
Impact of missing data on the internode distance matrix used by ASTRID. This figure shows the distance (denoted ∥*E*∥_2_) between the additive internode matrix *D*^*T*^ computed using the true species tree with unit branch lengths and the internode distance matrix *D*^*A*^ computed by ASTRID using true gene trees, as a function of the amount of missing data; see the main text for additional details. Each column represents a different level of incomplete lineage sorting (ILS): panel (**a**) shows datasets with low ILS, panel (**b**) shows datasets with high ILS, and panel (**c**) shows datasets with very high ILS. Lines represent the average over 20 replicate datasets, and filled regions indicate the standard error. Line color indicates the number of genes: datasets with 50, 200, and 1000 genes are shown in blue, orange, and green, respectively. Solid lines represent *M*_*iid*_ model of missing data, and dashed lines represent *M*_*clade*_ model of missing data. Note that datasets with 55 and 95% of genes with clade-based missing data had 34% and 59% total missing data, respectively. Datasets shown here have deep speciation events; results for datasets with estimated gene trees as well as recent speciation are shown in Additional file 1

As shown in Fig. 4, deleting taxa under the *M*_*iid*_ model resulted in an increase in the distance between *D*^*A*^ and *D*^*T*^; this is expected, since deleting taxa increases the variance and pairwise distances between leaves are either unchanged or are reduced. The impact of deleting taxa under the clade-based missing data models is quite different. First, taxon deletion typically reduced ∥*E*∥_2_; the only exception was for 50 genes with high levels of taxon deletion. Thus in general taxon deletion under clade-based models made the estimated internode distance matrix closer to additive.

**Experiment 2: Data requirements under the *M***_***iid***_** model of taxon deletion** We explored the performance of methods under conditions in which the three summary methods have been established to be statistically consistent (i.e., when taxa were deleted under the *M*_*iid*_ model and when true gene trees were given as input). We also provided true sequence alignments to SVDquartets, and so no species tree method had to handle estimation error in the inputs that they were given.

**Fig. 5:**
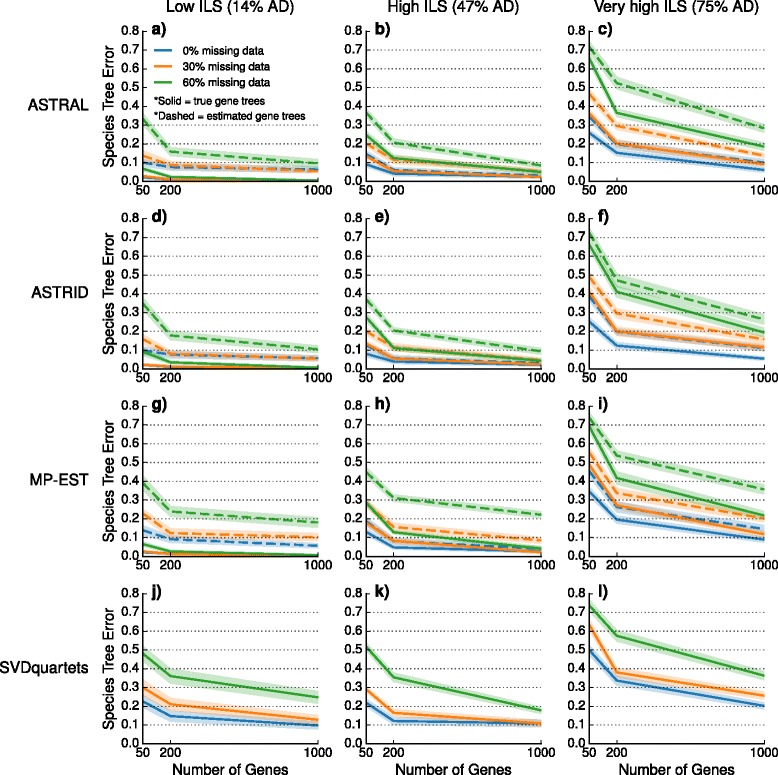
Species tree error for the *M*_*iid*_ model of missing data. Species tree error (measured by the RF error rate) is shown for different species tree estimation methods for increasing numbers of genes. Each column represents a different level of incomplete lineage sorting (ILS): panels (**a**), (**d**), (**g**), and (**j**) show datasets with low ILS, panels (**b**), (**e**), (**h**), and (**k**) show datasets with high ILS, and panels (**c**), (**f**), (**i**), and (**l**) show datasets with very high ILS. Each row represents a different species tree estimation method: panels (**a**)-(**c**) show ASTRAL, panels (**d**)-(**f**) show ASTRID, panels (**g**)-(**i**) show MP-EST, and panels (**j**)-(**l**) show SVDquartets. Lines represent the average over 20 replicate datasets, and filled regions indicate the standard error. Line color indicates the percentage of missing data: datasets with 0%, 30%, and 60% random missing data are shown in blue, orange, and green, respectively. Solid lines indicate that species tree estimation methods were given true gene trees, and dashed lines indicate that species tree estimation methods were given estimated gene trees. Note that SVDquartets was given the true multiple sequence alignment. Datasets shown here have deep speciation events; results for datasets with recent speciation are shown in Additional file 1

As shown in Fig. 5, species tree error rates decreased as the number of genes increases, under all ILS conditions. Under the lowest level of ILS (14% AD), all three summary methods had ∼10% or less error on average even when datasets had 60% missing data and only 50 genes. When ILS was high (47% AD), the summary methods required 200 genes to achieve an average error of less than 10% when there was 0% or 30% missing data and required 1000 genes to achieve a similar level of accuracy on datasets with 60% missing data. When datasets with the highest ILS condition (75% AD) had 60% missing data, all three summary methods approached ∼20% error on average when given 1000 genes. Hence, the data requirement for summary methods depended on both the percentage of missing data as well as other model conditions, such as the level of ILS. This was also true for SVDquartets. Although the error of species trees estimated by SVDquartets decreased substantially when given increasingly large numbers of genes, SVDquartets was much less accurate than the three summary methods (when given true genes) under all conditions, with largest differences for the lowest level of ILS.

These trends show that the data requirements for the summary methods are impacted by ILS level, so that accurate species tree estimation under higher levels of ILS requires a larger number of genes for all methods. This is intuitively obvious, as higher ILS results in greater heterogeneity between true gene trees, so that more gene trees are necessary to estimate the species tree (conversely, when there is no ILS, a single true gene tree suffices to estimate the species tree). These results are also consistent with [49], which examined the question theoretically when there are no missing data, establishing that the number of genes required by ASTRAL-II for accuracy with high probability is inversely proportional to the square of the length of the shortest branch in the species tree.

**Fig. 6:**
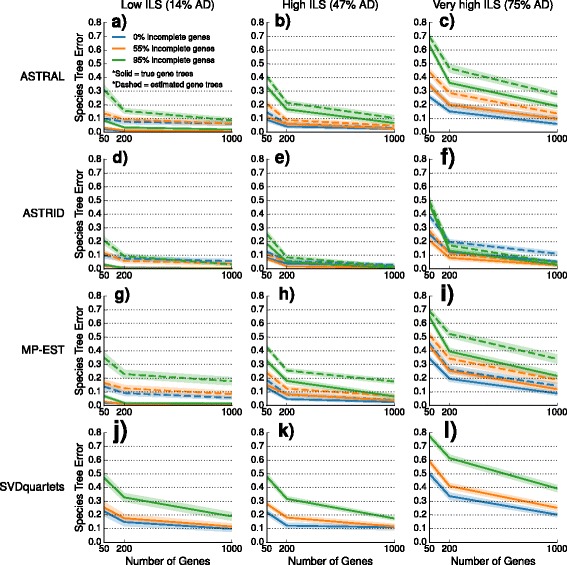
Species tree estimation error for the *M*_*clade*_ model of missing data. Species tree error (measured by the RF error rate) is shown for different species tree estimation methods for increasing numbers of genes. Each column represents a different level of incomplete lineage sorting (ILS): panels (**a**), (**d**), (**g**), and (**j**) show datasets with low ILS, panels (**b**), (**e**), (**h**), and (**k**) show datasets with high ILS, and panels (**c**), (**f**), (**i**), and (**l**) show datasets with very high ILS. Each row represents a different species tree estimation method: panels (**a**)-(**c**) show ASTRAL, panels (**d**)-(**f**) show ASTRID, panels (**g**)-(**i**) show MP-EST, and panels (**j**)-(**l**) show SVDquartets. Lines represent the average over 20 replicate datasets, and filled regions indicate the standard error. Line color indicates the percentage of missing data: datasets with 0%, 30%, and 60% clade-based missing data are shown in blue, orange, and green, respectively. Solid lines indicate that species tree estimation methods were given true gene trees, and dashed lines indicate that species tree estimation methods were given estimated gene trees. Note that SVDquartets was given the true multiple sequence alignment. Datasets shown here have deep speciation events; results for datasets with recent speciation are shown in Additional file 1

**Experiment 3: Data requirements under the *M***_***clade***_** model of taxon deletion** We explored the performance of methods when taxa were deleted from genes under the *M*_*clade*_ model of missing data. As with Experiment 2, we provided true gene trees as input to the summary methods and true gene sequence alignments as input to SVDquartets. Results in this experiment (Fig. 6) were nearly identical to the results under the *M*_*iid*_ model, except that ASTRID had much lower error than the other methods (Fig. 6d-f). In fact, the accuracy of ASTRID sometimes improved with increasing percentages of genes with clade-based missing data; this was not true for increasing values of *p* for the *M*_*iid*_ model of missing data. The difference between ASTRAL and ASTRID under the clade-based model of taxon deletion is interesting and provocative.

**Experiment 4: Data requirements given estimated gene trees** We explored the performance of species tree estimation methods under both models of taxon deletion, using estimated gene trees rather than true gene trees for summary methods and true multiple sequence alignments for SVDquartets. Hence, the results for the summary methods were different for this experiment than for the previous two experiments, while the results for SVDquartets were the same as for the previous two experiments.

**Fig. 7:**
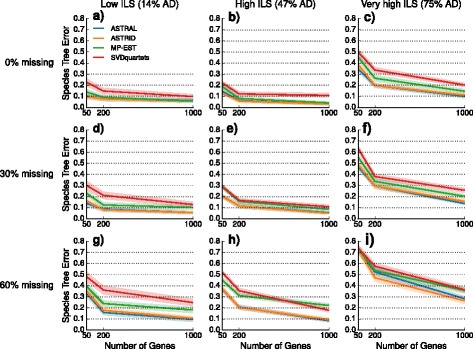
Comparison of species tree estimation methods given estimated gene trees for the *M*_*iid*_ model of missing data. Species tree estimation error (measured by the RF error rate) is shown for species trees estimated by giving increasing numbers of genes as inputs to ASTRAL (blue), ASTRID (orange), MP-EST (green), and SVDquartets (red). All three summary methods were given estimated gene trees, and SVDquartets was given the true multiple sequence alignment. Each column represents a different level of incomplete lineage sorting (ILS): panels (**a**), (**d**), and (**g**) show datasets with low ILS, panels (**b**), (**e**), and (**h**) show datasets with high ILS, and panels (**c**), (**f**), and (**i**) show datasets with very high ILS. Each row represents a different percentage of missing data: panels (**a**)-(**c**) show datasets with 0% missing data, panels (**d**)-(**f**) show datasets with 30% random missing data, and panels (**g**)-(**i**) show datasets with 60% random missing data. Lines represent the average over 20 replicate datasets, and filled regions indicate the standard error. Datasets shown here have deep speciation events; results for datasets with recent speciation are shown in Additional file 1

**Fig. 8:**
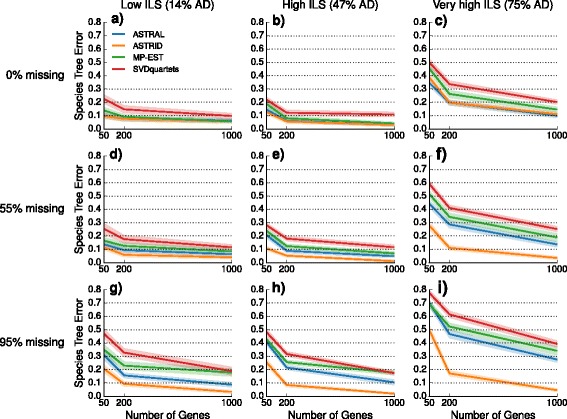
Comparison of species tree estimation methods given estimated gene trees for the *M*_*clade*_ model of missing data. Species tree error (defined as the RF error rate) is shown for species trees estimated by giving increasing numbers of genes as inputs to ASTRAL (blue), ASTRID (orange), MP-EST (green), and SVDquartets (red). All three summary methods were given estimated gene trees, and SVDquartets was given the true multiple sequence alignment. Each column represents a different level of incomplete lineage sorting (ILS): panels (**a**), (**d**), and (**g**) show datasets with low ILS, panels (**b**), (**e**), and (**h**) show datasets with high ILS, and panels (**c**), (**f**), and (**i**) show datasets with very high ILS. Each row represents a different percentage of genes with missing data: panels (**a**)-(**c**) show datasets with 0% of genes missing data, panels (**d**)-(**f**) show datasets with 55% of genes missing data, and panels (**g**)-(**i**) show datasets with 95% of genes missing data. For example, if 55% of the 1000 genes are missing data, then 450 genes are complete and 550 genes are incomplete (due to clade-based missing data). Lines represent the average over 20 replicate datasets, and filled regions indicate the standard error. Datasets shown here have deep speciation events; results for datasets with recent speciation are shown in Additional file 1

Results on estimated gene trees are shown in Fig. 7 for the *M*_*iid*_ taxon deletion model and Fig. 8 for the clade-based taxon deletion model. Overall these results are largely similar to the results when species tree methods are given true gene trees (i.e., compare to Figs. 5 and 6). For example, error rates dropped as the number of genes increased and error rates increased with the level of ILS. Unsurprisingly, overall accuracy was reduced for summary methods when given estimated as opposed to true gene trees under both models. Also, the relative performance between methods was largely the same as when using true gene trees, with a few differences. On true gene trees all three summary methods had very close accuracy, but on estimated gene trees, ASTRAL and ASTRID were generally more accurate than MP-EST, with ASTRID having a clear advantage over ASTRAL under clade-based missing data. The gap between SVDquartets and the summary methods also narrowed, so that although SVDquartets remained less accurate than ASTRAL and ASTRID, on occasion it matched or exceeded the accuracy of MP-EST for large enough numbers of genes (e.g., see Fig. 7h).

## Discussion

Our study provides theoretical results, establishing consistency of summary methods under some models of taxon deletion, as well as new experimental results. The theoretical results are novel, and to our knowledge are the first such results.

The results of our simulation study can be compared to prior simulation studies [7, 23–25] that also examined the impact of missing data on coalescent-based species tree estimation methods using simulated datasets with gene tree heterogeneity due to ILS. These studies differed in terms of species tree estimation methods, models of missing data, and other conditions, including ILS levels, sequence evolution models, deviation from a strict molecular clock, and gene tree estimation error; our study is closest to [24] in that we evaluated the same species tree estimation methods.

All these simulation studies found that good species tree accuracy could be obtained for the better methods when enough genes were available; however, some of these prior studies explored conditions that may have improved the robustness of methods to missing data (e.g., [23, 25] simulated sequence evolution under a strict molecular clock; [23] never deleted the outgroup taxon from gene alignments, and so all estimated gene trees could be rooted using the outgroup before being given to MP-EST, which requires rooted gene trees). None of these prior studies explored performance under the two models of missing data we examine (i.e., the *i.i.d.* model and the clade-based missing data model), and none examined the impact of missing data when given true gene trees, which is a main focus of this study.

Our findings are consistent with the trends shown in the prior studies, demonstrating that ASTRAL-II, ASTRID, MP-EST, and SVDquartets can achieve good accuracy when given a large enough number of genes. As expected, the summary methods have the best accuracy when given true gene trees, but good results are also obtained when using a sufficient number of estimated gene trees for the conditions we examined. Under both models of missing data, the three summary methods are more accurate than SVDquartets even when given estimated gene trees, and ASTRAL-II and ASTRID have better accuracy than MP-EST.

The analyses under clade-based models of missing data are novel, as prior studies did not examine this model. Furthermore, ASTRID substantially outperformed the other methods, and even improved in accuracy under some conditions, when taxa were deleted under a clade-based model. However, the other species tree estimation methods all degraded in accuracy under the clade-based model.

To understand the difference in how ASTRID responded to clade-based missing data compared to the other summary methods, we examined how taxon deletion impacted the input to the summary methods (i.e., the gene trees and the internode distance matrix). As shown in Fig. 3, GTEE and AD values increased under *M*_*iid*_ models but decreased under *M*_*clade*_ models of taxon deletion; furthermore, Fig. 4 shows that ∥*E*∥_2_ (a measure of the distance between the internode distance matrix defined by the model species tree and the internode distance matrix that ASTRID computes) increased under *M*_*iid*_ models but generally decreased under *M*_*clade*_ models. These results suggest that summary methods should become less accurate under *M*_*iid*_ models (a trend we observe in our simulation study), but also suggest that summary methods should become more accurate under the *M*_*clade*_ models. However, only ASTRID became more accurate under *M*_*clade*_ models. Furthermore, the conditions where ASTRID became more accurate as taxa were deleted under *M*_*clade*_ models were generally those that resulted in the distance between *D*^*A*^ and *D*^*T*^ decreasing.

One possible explanation for why taxon deletion under the clade-based models did not result in improved accuracy for ASTRAL and MP-EST summary methods, despite the reduction in GTEE and AD, is that taxon deletion also automatically reduces the amount of information available, and that reduction in information quantity has a larger negative impact on ASTRAL and MP-EST than the positive impact obtained by the improvement in information quality (as indicated by the reduction in GTEE and AD values). This trade-off between information quality and quantity seems to be different for ASTRID than for ASTRAL and MP-EST, however, since ASTRID typically benefits under clade-based models (when there are enough genes).

Thus, ASTRID responds differently from other species tree estimation methods, and these differences suggest that the pattern of missing data in an empirical dataset may have implications for selecting summary methods for phylogenomic species tree estimation. In particular, if the data that are gathered seem to have missing data patterns that look clade-based, then ASTRID may be preferable to ASTRAL and MP-EST, while if the data that are gathered seem to have other types of missing data patterns then ASTRAL may be preferable.

## Conclusions

Species tree estimation using multi-locus datasets is increasingly common, but many datasets have substantial numbers of incomplete gene trees. Furthermore, many species tree estimation methods have been shown to reduce in accuracy as taxa are deleted from gene trees, leading to the concern that species tree estimation methods might not be statistically consistent under models of taxon deletion.

This study proved that many standard species tree estimation methods remain statistically consistent under the MSC model under some simple models of missing data. However, we also proved that under some more complex models (and in particular under an adversary model) of missing data, some popular species tree methods can be inconsistent, indicating that the frequency and type of missing data are important factors in determining the impact of missing data on species tree estimation.

Our study also examined the accuracy of species trees estimated on finite numbers of genes using both true and estimated gene trees. We showed that a high degree of accuracy is achievable using four common species tree methods (ASTRAL-II, ASTRID, MP-EST, and SVDquartets) when given a large enough number of genes, even when there is a high degree of missing data, gene tree heterogeneity resulting from ILS, and moderate levels of gene tree estimation error. We also saw some differences between methods that depended on the type and degree of missing data, with ASTRAL typically having among the best accuracy of all methods; however, under clade-based models of missing data, ASTRID was usually the most accurate.

Our theory and experiments are suggestive of why taxon deletion under *M*_*iid*_ models generally reduces accuracy for all species tree estimation methods we explored, and also why taxon deletion under clade-based models improves accuracy for ASTRID; however, further research is needed to understand other trends we observe in these experiments. For example, we do not know why taxon deletion under the clade-based models reduces gene tree estimation error and ILS levels (as measured using AD values), nor why ASTRAL does not improve under clade-based taxon deletion models although ASTRID does. Several interesting mathematical questions also remain unanswered. Very little is known about data requirements for coalescent-based species tree estimation, even when all genes are complete (although see [49] for bounds on the number of true gene trees needed for species tree accuracy with high probability for ASTRAL-II). This study also showed that species tree estimation accuracy was impacted by missing data and also by gene tree estimation error. Since many real world datasets have both incomplete gene trees and high gene tree estimation error (see discussion in [24]), perhaps the most important theoretical question is whether statistically consistent species tree estimation using summary methods is possible in the presence of both these conditions. However, very little is known about this, even when there are no missing data [33].

The choice of method for species tree estimation method depends on a combination of factors, including statistical properties (such as statistical consistency) and performance on simulated and biological datasets. Our findings are generally positive, showing substantial robustness to taxon deletion in terms of empirical performance for several popular species tree estimation methods, and establishing that statistical consistency can still be guaranteed for some popular methods under some models of taxon deletion (in some cases, by simple modifications to the method). Thus, our study shows that statistically consistent methods that are highly accurate exist for species tree estimation even in the presence of missing data and gene tree heterogeneity due to incomplete lineage sorting. Overall, this study adds to a growing body of literature addressing the impact of missing data on species tree estimation, and supports the conclusion that missing data - in itself - is not particularly problematic for species tree estimation.

## Additional file

Additional file 1 Supplementary Materials. Detailed methods and commands for tree estimation and evaluation. Eight (8) figures and three (3) tables describing additional results, including those for simulated datasets when species trees were simulated with recent speciation. (PDF 489 kb)

## Abbreviations

AD: Normalized Robinson-Foulds distance between the true species and the true gene trees averaged over all gene trees or “average distance”
GTEE: Gene tree estimation error
*i.i.d.*: Independent and identically distributed
ILS: Incomplete lineage sorting
MSC: Multi-species coalescent
RF: Robinson-Foulds distance
UCE: Ultra-conserved elements
