# Correction to: The performance of coalescent-based species tree estimation methods under models of missing data

**DOI:** 10.1186/s12864-020-6540-1

**Published:** 2020-02-10

**Authors:** Michael Nute, Jed Chou, Erin K. Molloy, Tandy Warnow

**Affiliations:** 1 Department of Statistics, University of Illinois at Urbana-Champaign, 725 S. Wright St., Champaign, IL 61820 USA; 2 Department of Mathematics, University of Illinois at Urbana-Champaign, 1409 W. Green St., Urbana, IL 61801 USA; 3 Department of Computer Science, University of Illinois at Urbana-Champaign, 201 North Goodwin Avenue, Urbana, IL 61801 USA

**Correction to: BMC Genomics**

**https://doi.org/10.1186/s12864-018-4619-8**

After publication of [1], the authors were informed by John A. Rhodes of a counterexample to Theorem 11 of [1]. The counterexample and its consequences with respect to the theoretical properties of NJst [2] and ASTRID [3] are provided in [4] and summarized here. The authors of [1] apologize for the mistake in the proof.

The question of interest in [1] is whether several species tree estimation methods that operate by combining gene trees (e.g., ASTRAL [5], ASTRID [3], and NJst [2]) remain statistically consistent when data are missing due to random taxon deletion, under the assumption that the gene trees are generated by the multi-species coalescent (MSC) model [6] and so can differ from the true species tree due to incomplete lineage sorting (ILS). Theorem 11 addresses this issue for NJst and ASTRID with the *M*_*iid*_ model of taxon deletion, which assumes that taxa are deleted independently and identically from the gene trees. NJst and ASTRID estimate the species tree in two steps. In the first step, each calculates the internode distance matrix (of average pairwise distances between species, computed from the gene trees), and in the second step each computes a tree from the distance matrix using either neighbor joining [7] or balanced minimum evolution (BME) with FastME [8], respectively.

Furthermore, neighbor joining and FastME are both guaranteed to return a tree *T* when given a matrix that is sufficiently close to an additive matrix for *T* (where a matrix *A* is additive for *T* if the edges of *T* can be assigned non-negative lengths so that for all *i, j*, *A*_*ij*_ is the sum of the edge lengths in the path from *i* to *j* in *T*) [9]. While it is established that the internode distance matrix converges to an additive matrix for the species tree if there is no taxon deletion [10], it was not known if it converged to an additive distance matrix in the presence of taxon deletion. In the attempted proof of Theorem 11, Nute et al. argued that the internode distance matrix computed for gene trees that evolve under the MSC and then have taxa deleted under the *M*_*iid*_ model converges to an additive matrix for the species tree.

Were their argument correct, then both NJst and ASTRID would be statistically consistent under the combination of the MSC and *M*_*iid*_ models, which is what Theorem 11 of [1] claims. However, Rhodes et al. [4] presented an example of a model species tree and taxon deletion probability so that the internode distance matrix does not converge, as the number of genes increases, to a matrix that is additive for the model species tree topology. Furthermore, they prove that as the number of gene trees increases, NJst and ASTRID will converge to a tree other than the true species tree. Therefore, neither NJst nor ASTRID are statistically consistent under the combination of MSC and *M*_*iid*_ taxon deletion, and in fact are positively misleading. Here we describe the counterexample from [4] and sketch the proof that shows that Theorem 11 is incorrect; the details of the proof that ASTRID and NJst are not statistically consistent under the MSC + *M*_*iid*_ model are available in [4].

Consider the balanced ultrametric species tree on six taxa *a, b, c, d, e, f*

*σ* = ((*a*: *L* + 1*,* (*b*: 1*, e*: 1): *L*): *E,* (*c*: *L* + 1*,* (*d*: 1*, f*: 1): *L*): *E*)*,*

where *E* and *L* are measured in coalescent units. Rhodes et al. [4] showed that when *L* = *∞*, *E* = 0, and *p ∈* (0*,* 1) (where *p* gives the probability of taxon presence under *M*_*iid*_), the expected internode distance matrix under the combined *MSC* + *M*_*iid*_ model is additive for a tree with a topology different from *σ*; in particular, it will display quartet tree (*ac, bd*) (which is the tree with the leaves for *a, c* separated from the leaves for *b, d* by one or more edges) whereas *σ* displays (*ab, cd*).

Therefore, by continuity of the expected distances, when *E >* 0 is sufficiently small and *L* is finite but sufficiently large, the expected distance matrix will be sufficiently close to the additive matrix inducing quartet tree (*ac, bd*) that both neighbor joining and BME within FastME will return a tree that displays (*ac, bd*).

In summary, [4] provides a construction of binary model species trees with finite edge lengths (in coalescent units) on which the expected internode distance matrix will be close to an additive matrix for a tree other than the model species tree, and NJst and ASTRID will converge to a tree other than the model species tree, thus establishing that Theorem 11 in [1] is incorrect. We note that [4] did not provide counterexamples for any theorem regarding statistical consistency for ASTRAL under models of missing data, so the counterexample in [4] is applicable to only NJst and ASTRID.
