# Supplementary material for: The performance of coalescent-based species tree estimation methods under models of missing data
Source: BMC Genomics. 2018 May 8;19(Suppl 5):286. doi: 10.1186/s12864-018-4619-8 (PMC5998899; doi:10.1186/s12864-018-4619-8)
Supplement: Supplementary file 1 — Supplementary Materials. Detailed methods and commands for tree estimation and evaluation. Eight (8) figures and three (3) tables describing additional results, including those for simulated datasets when species trees were simulated with recent speciation. (PDF 489 kb) [file 12864_2018_4619_MOESM1_ESM.pdf]

The performance of coalescent-based species tree estimation methods under models of missing data

Supplementary Materials

Michael Nute, Jed Chou, Erin K. Molloy, and Tandy Warnow

April 5, 2018

Contents

List of Figures 1

List of Tables 1

1 Methods 2
- 1.1 Simulated Datasets and Gene Tree Estimation 2
- 1.2 Species Tree Estimation 3

2 Results 5

References 13

List of Figures

S1 Impact of missing data on AD and GTEE values (recent speciation) 5
S2 Species tree error for the M_{iid} model of missing data (recent speciation). 6
S3 Species tree estimation error for the M_{clade} model of missing data (recent speciation) 7
S4 Impact of missing data on ASTRID, given estimated gene trees (deep speciation) 8
S5 Impact of missing data on ASTRID, given true gene trees (recent speciation) 9
S6 Impact of missing data on ASTRID, given estimated gene trees (recent speciation) 10
S7 Comparison of species tree estimation methods given estimated gene trees for the M_{iid} model of missing data (recent speciation) 11
S8 Comparison of species tree estimation methods given estimated gene trees for the M_{clade} model of missing data (recent speciation) 12

List of Tables

S1 Simulated gene trees 2
S2 Gene sampling for datasets with clade-based missing da ta 3
S3 Commands for species tree methods 4

1 Methods

1.1 Simulated Datasets and Gene Tree Estimation

Gene trees were estimated using RAxML v. 8.2.8 with the following command:

```
raxmlHPC−SSE3 −m GTRGAMMA
              −p [seed]
              −n [output_name]
              −s [alignment_file]
```

As MP-EST requires rooted gene trees, gene trees were rooted using Dendropy 4.3.0 (Sukumaran and Holder, 2010). When available, gene trees were rooted at their outgroup:

```
outgroup = g.find_node_with_taxon_label([outgroup_name])
e = outgroup.edge.length
g.reroot_at_edge(outgroup.edge, update_bipartitions=False)
outgroup.edge.length = e / 2.0
```

Otherwise, incomplete gene trees were rooted at the midpoint of the longest leaf-to-leaf path:

```
[gene_tree].reroot_at_midpoint(update_bipartitions=False)}
```

Level of ILS	Species Tree Height	Average Distance	Mean Gene Tree Error	Total Discord
Deep speciation (rate: 10^{-7})				
Low ILS	10M	0.14 ± 0.01	0.44 ± 0.14	0.46 ± 0.13
High ILS	2M	0.47 ± 0.02	0.40 ± 0.09	0.59 ± 0.06
Very high ILS	500K	0.75 ± 0.01	0.44 ± 0.17	0.81 ± 0.04
Recent speciation (rate: 10^{-6})				
Low ILS	10M	0.10 ± 0.01	0.22 ± 0.10	0.26 ± 0.09
High ILS	2M	0.35 ± 0.02	0.34 ± 0.12	0.49 ± 0.08
Very high ILS	500K	0.75 ± 0.01	0.49 ± 0.18	0.82 ± 0.05

Table S1: **Simulated gene trees for 1000-gene datasets with no missing data.** Average distance is the normalized Robinson-Foulds or RF distance between the true species tree and true gene trees, averaged across all genes; mean gene tree error is the normalized RF distance between true gene trees and estimated gene trees averaged across all genes; total discord is the normalized RF distance between the true species tree and estimated gene trees averaged across all genes. Means (± standard deviations) are across 20 replicate datasets for each model condition.

1.2 Species Tree Estimation

Species trees were estimated using ASTRAL (Mirarab and Warnow, 2015), ASTRID (Vachaspati and Warnow, 2015), MP-EST (Liu et al., 2010), and SVDquartets (Chifman and Kubatko, 2014, 2015) within PAUP* (Swofford, 2003); see Table S3 for details. Species trees were estimated sampling the first 50, 200, and 1000 genes in each datasets. When datasets had clade-based missing data, complete/incomplete genes were sampled as shown in Table S2.

	55% Incomplete Genes		95% Incomplete Genes	
	Complete	Incomplete	Complete	Incomplete
50 genes	1-22	23-50	1-2	3-50
200 genes	1-90	91-200	1-20	11-200
1000 genes	1-450	451-1000	1-50	51-1000

Table S2: **Gene sampling for datasets with clade-based missing data.** The gene numbers that were sampled from datasets with complete genes and datasets with incomplete genes (with clade-based missing data) to create datasets with 55% and 95% of genes being incomplete due to clade-based missing data. For example, to create a 50-gene dataset with 95% clade-based missing data, gene 1 and gene 2 were taken to be complete (no missing data) and the remaining genes (labeled 3-50) were taken to be incomplete (clade-based missing data).

SVDquartets failed to complete on one dataset (number of genes: 50, species tree height: 500K, speciation rate: 1E-6, replicate number: 4, 60% *i.i.d.* missing data) with the error message: "No informative quartets were found in SVDQuartets analysis".

Species tree error was reported as the normalized Robinson-Foulds (RF) distance (Robinson and Foulds, 1981), computed using a custom python script:

```
def compare_trees(tr1, tr2):
    from dendropy.calculate.treecompare \
        import false_positives_and_negatives

    lb1 = [l.taxon.label for l in tr1.leaf_nodes()]
    lb2 = [l.taxon.label for l in tr2.leaf_nodes()]

    com = list(set(lb1).intersection(lb2))
    tns = dendropy.TaxonNamespace(com)

    tr1.retain_taxa_with_labels(com)
    tr1.migrate_taxon_namespace(tns)
    tr1.update_bipartitions()

    tr2.retain_taxa_with_labels(com)
    tr2.migrate_taxon_namespace(tns)
    tr2.update_bipartitions()

    ei1 = len(tr1.internal_edges(exclude_seed_edge=True))
    ei2 = len(tr2.internal_edges(exclude_seed_edge=True))

    nl = len(com)
    [fp, fn] = false_positives_and_negatives(tr1, tr2)
    rf = float(fp + fn) / ((nl - 3) * 2)

    return(nl, ei1, ei2, fp, fn, rf)
```

This script ensures that the two trees being compared are first constrained to the same set of taxa.

Method	Version	Command	Notes
ASTRAL	4.10.5	`java -jar astral.4.10.5.jar -i [gene_trees_file] -o [output_file]`	Download: `github.com/smirarab/astral`
ASTRID	1.1	`ASTRID -i [gene_trees_file] -o [output_file]`	Download: `github.com/pranjalv123/ASTRID`
MP-EST	1.5	`mpest [control_file]`	Control file specified 10 independent runs with random starting trees.<br><br>Download: `faculty.franklin.uga.edu/lliu/content/mp-est`
SVDquartets	4a154	`echo ''exe [alignment_file]; svd showScores=no evalQuartets=all qformat=qmc replace=no; savetrees file=[output_file] format=newick;'' | paup4a150_centos64 -n`	Download: `people.sc.fsu.edu/~dswofford/paup_test`

Table S3: **Information about commands used to estimate species trees.**

2 Results

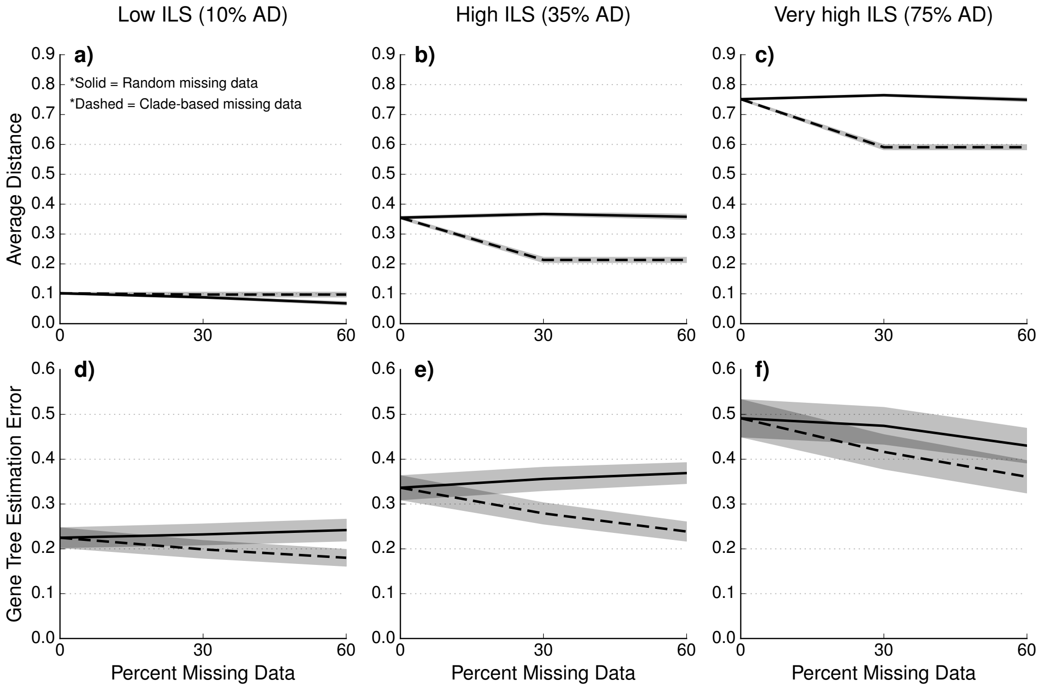

Figure S1: **Impact of missing data on AD and GTEE values (recent speciation).** Average distance (or AD, defined as the normalized Robinson-Foulds or RF distance between the true species tree and the true gene trees, averaged across all 1000 genes) and gene tree estimation error (or GTEE, defined as the normalized RF distance between the true and the estimated gene trees, average across all 1000 genes) are shown for increasing amounts of missing data. Each column represents a different level of incomplete lineage sorting (ILS). Lines represent the average over 20 replicate datasets, and filled regions indicate the standard error. Solid lines and dashed lines represent the M_{iid} and M_{clade} models of missing data, respectively. Note that datasets with 55% and 95% of genes with clade-based missing data had 34% and 59% total missing data, respectively. Datasets shown here have recent speciation events and 1000 genes; results for datasets with deep speciation are shown in the main paper.

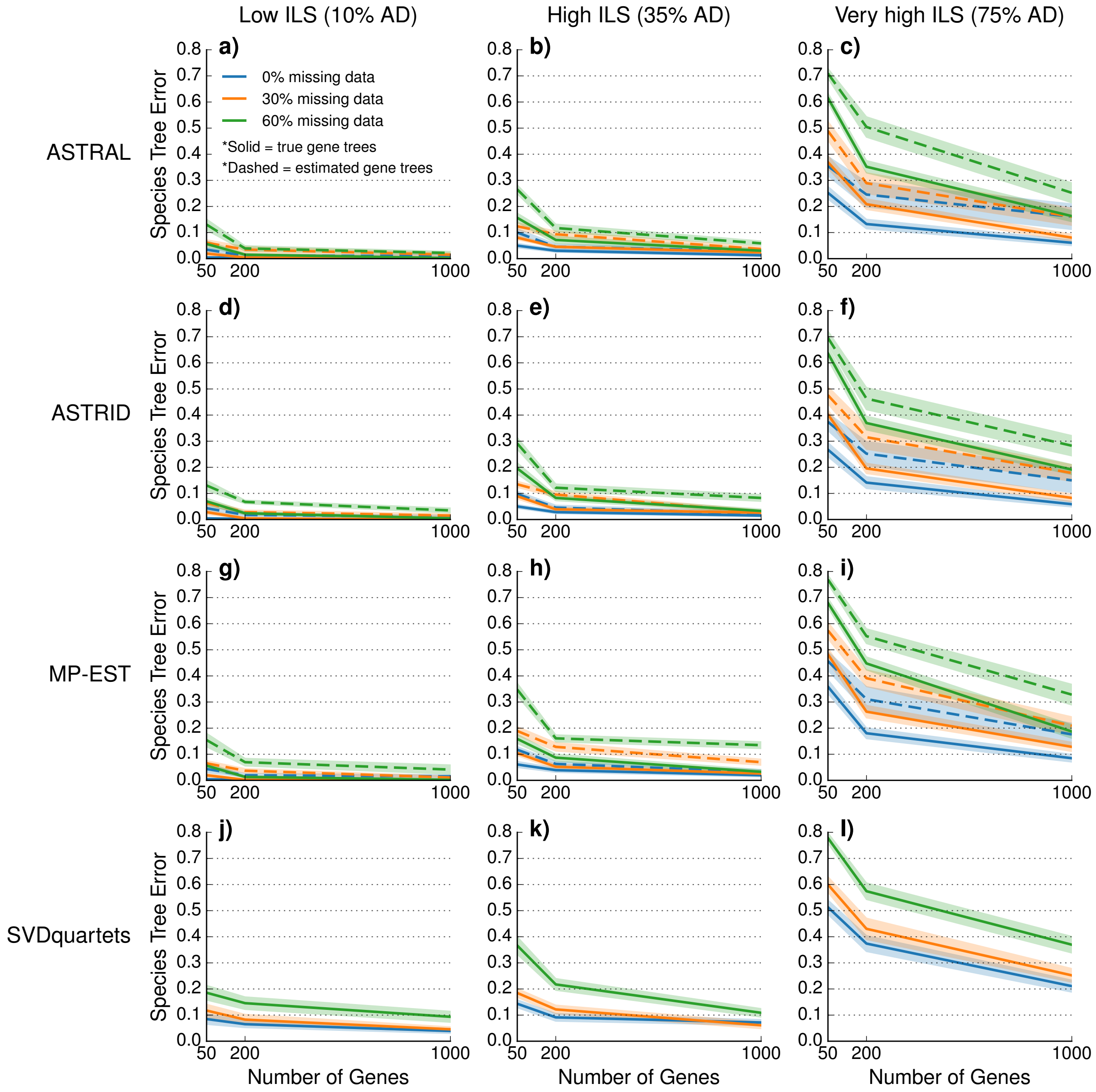

Figure S2: **Species tree error for the M_{iid} model of missing data (recent speciation).** Species tree error (measured by the RF error rate) is shown for increasing numbers of genes. Each column represents a different level of incomplete lineage sorting (ILS), and each row represents a different species tree estimation method. Lines represent the average over 20 replicate datasets, and filled regions indicate the standard error. Line color indicates the percentage of missing data: datasets with 0%, 30%, and 60% random missing data are shown in blue, orange, and green, respectively. Solid lines indicate that species trees were estimated from true gene trees, and dashed lines indicate that species tree were estimated from estimated gene trees. Note that SVDquartets was estimated using the tree multiple sequence alignment. Datasets shown here had recent speciation events; results for datasets with deep speciation events are shown in the main paper.

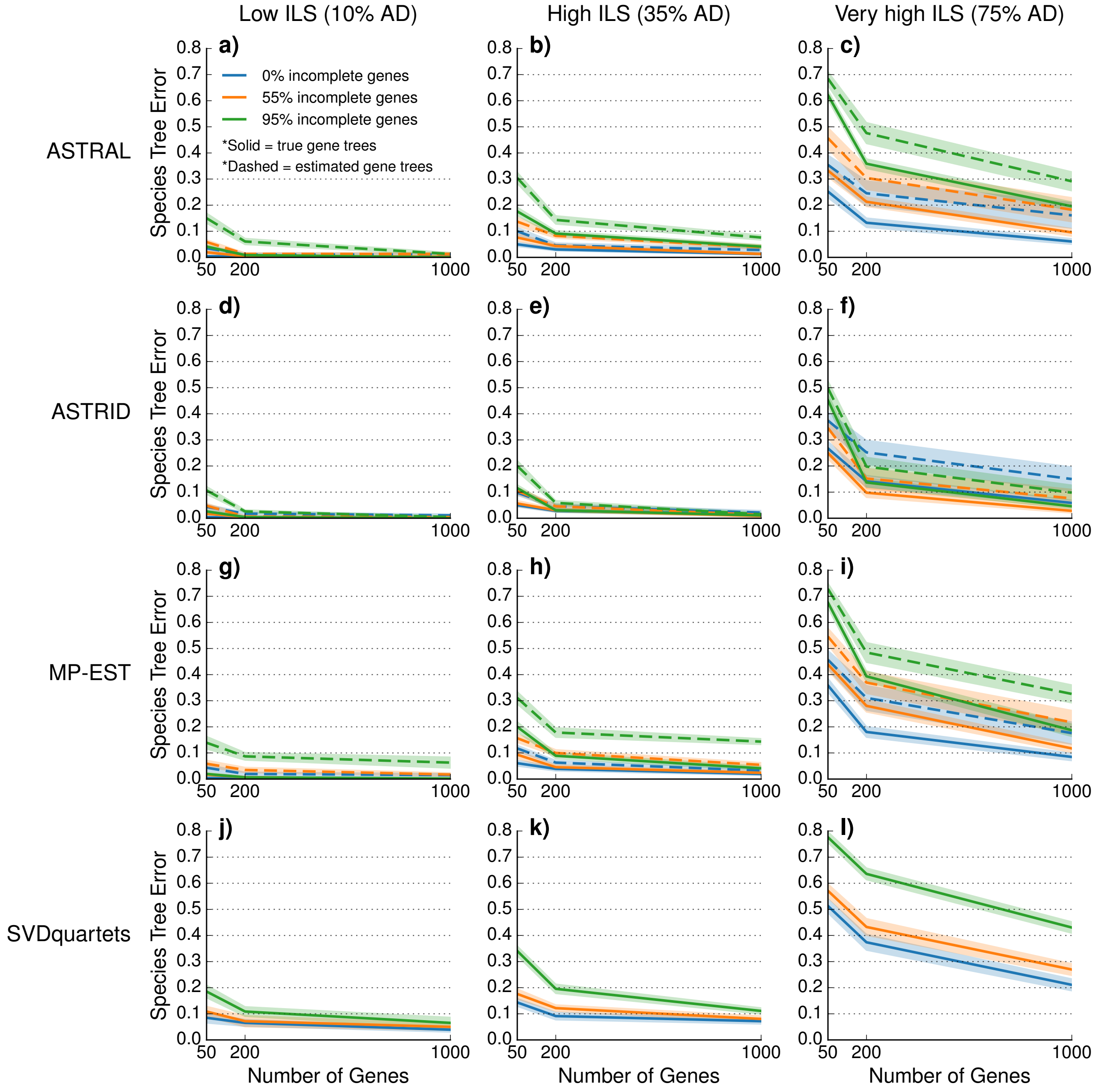

Figure S3: **Species tree estimation error for the M_{clade} model of missing data (recent speciation).** Species tree error (measured by the RF error rate) is shown for increasing numbers of genes. Each column represents a different level of incomplete lineage sorting (ILS), and each row represents a different species tree estimation method. Lines represent the average over 20 replicate datasets, and filled regions indicate the standard error. Line color indicates the percentage of missing data: datasets with 0%, 30%, and 60% clade-based missing data are shown in blue, orange, and green, respectively. Solid lines indicate that species trees were estimated from true gene trees, and dashed lines indicate that species tree were estimated from estimated gene trees. Note that SVDquartets was estimated using the tree multiple sequence alignment. Datasets shown here had recent speciation events; results for datasets with deep speciation events are shown in the main paper.

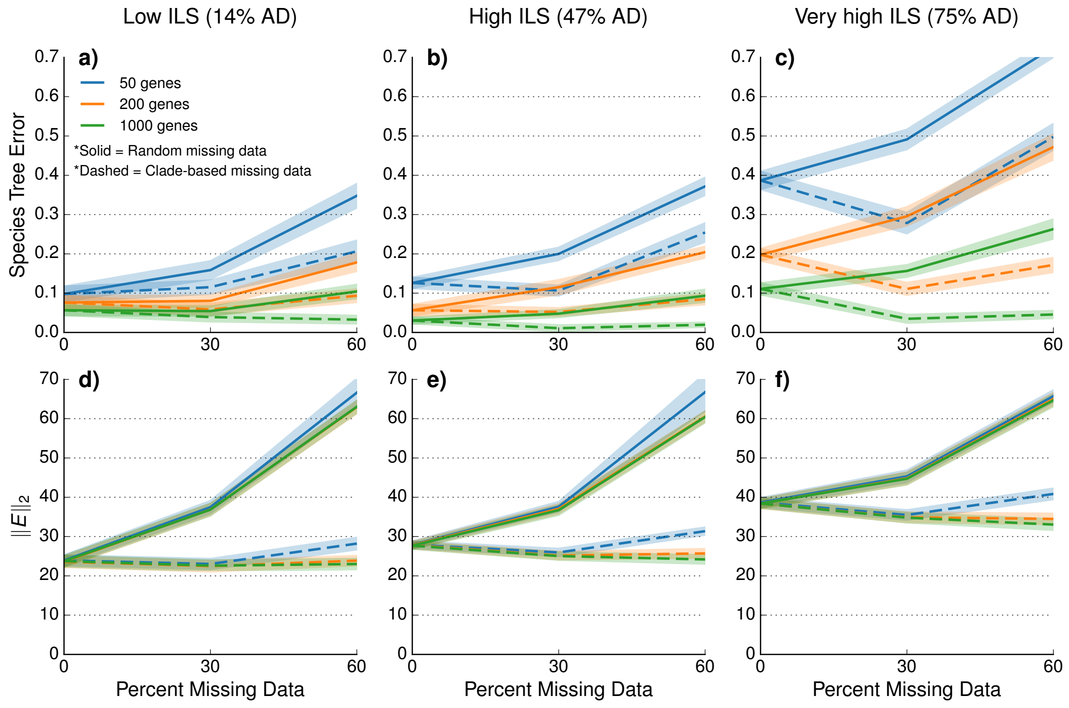

Figure S4: **Impact of missing data on ASTRID, given estimated gene trees (deep speciation).** The top row shows the species tree error for the ASTRID tree computed using estimated gene trees, and the bottom row shows the L_2 distance (denoted $\|E\|_2$) between the additive matrix computed using the true species tree with unit branch lengths and the internode distance matrix computed by ASTRID using estimated gene trees. These two metrics are shown for increasing amounts of missing data. Each column represents a different level of incomplete lineage sorting (ILS). Lines represent the average over 20 replicate datasets, and filled regions indicate the standard error. Line color indicates the number of genes: datasets with 50, 200, and 1000 genes are shown in blue, orange, and green, respectively. Solid lines represent M_{iid} model of missing data, and dashed lines represent M_{clade} model of missing data. Note that datasets with 55% and 95% of genes with clade-based missing data had 34% and 59% total missing data, respectively. Datasets shown here have deep speciation events.

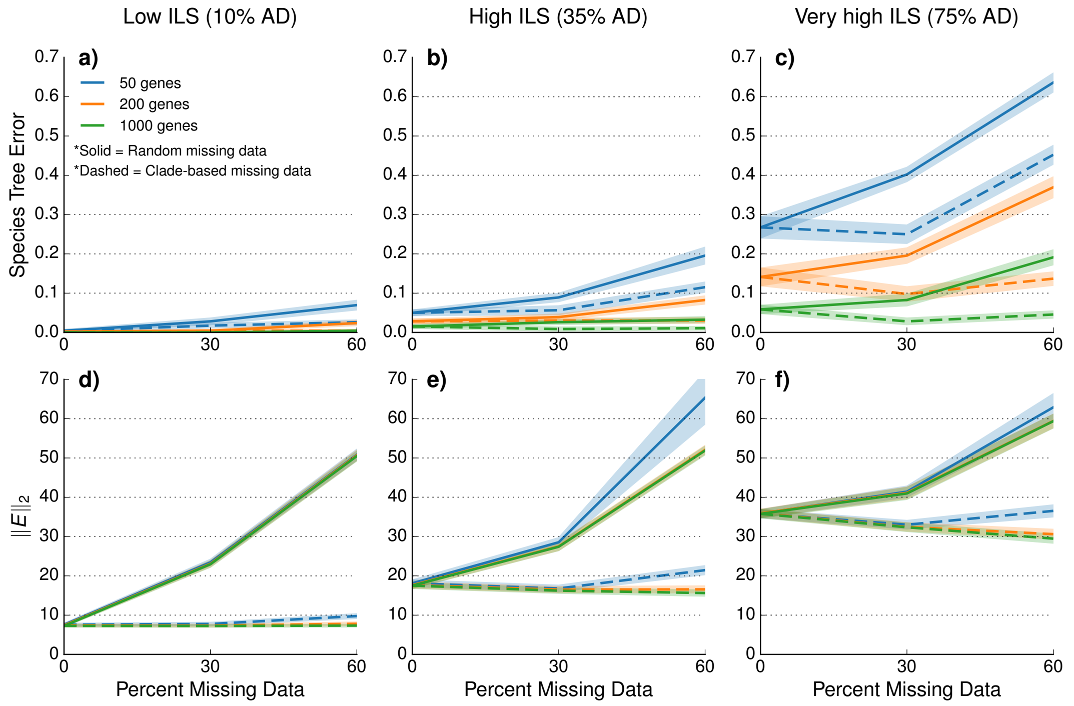

Figure S5: **Impact of missing data on ASTRID, given true gene trees (recent speciation).** The top row shows the species tree error for the ASTRID tree computed using true gene trees, and the bottom row shows the L_2 distance (denoted $\|E\|_2$) between the additive matrix computed using the true species tree with unit branch lengths and the internode distance matrix computed by ASTRID using true gene trees. These two metrics are shown for increasing amounts of missing data. Each column represents a different level of incomplete lineage sorting (ILS). Lines represent the average over 20 replicate datasets, and filled regions indicate the standard error. Line color indicates the number of genes: datasets with 50, 200, and 1000 genes are shown in blue, orange, and green, respectively. Solid lines represent M_{iid} model of missing data, and dashed lines represent M_{clade} model of missing data. Note that datasets with 55% and 95% of genes with clade-based missing data had 34% and 59% total missing data, respectively. Datasets shown here have recent speciation events.

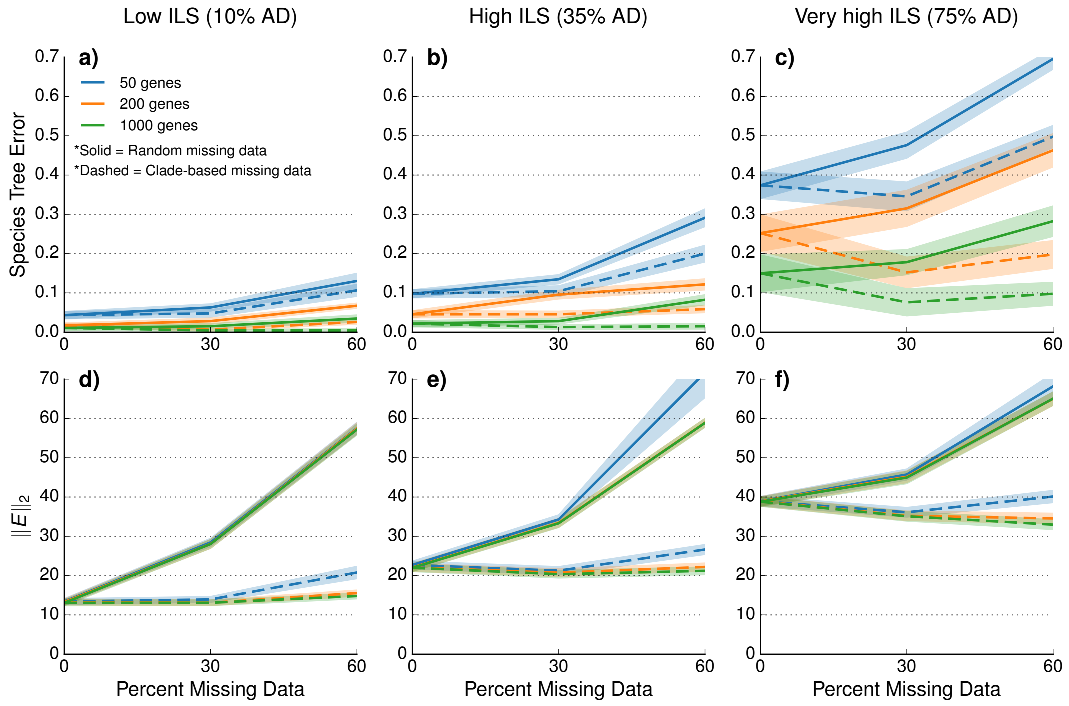

Figure S6: **Impact of missing data on ASTRID, given estimated gene trees (recent speciation).** The top row shows the species tree error for the ASTRID tree computed using estimated gene trees, and the bottom row shows the L_2 distance (denoted $\|E\|_2$) between the additive matrix computed using the true species tree with unit branch lengths and the internode distance matrix computed by ASTRID using estimated gene trees. These two metrics are shown for increasing amounts of missing data. Each column represents a different level of incomplete lineage sorting (ILS). Lines represent the average over 20 replicate datasets, and filled regions indicate the standard error. Line color indicates the number of genes: datasets with 50, 200, and 1000 genes are shown in blue, orange, and green, respectively. Solid lines represent M_{iid} model of missing data, and dashed lines represent M_{clade} model of missing data. Note that datasets with 55% and 95% of genes with clade-based missing data had 34% and 59% total missing data, respectively. Datasets shown here have recent speciation events.

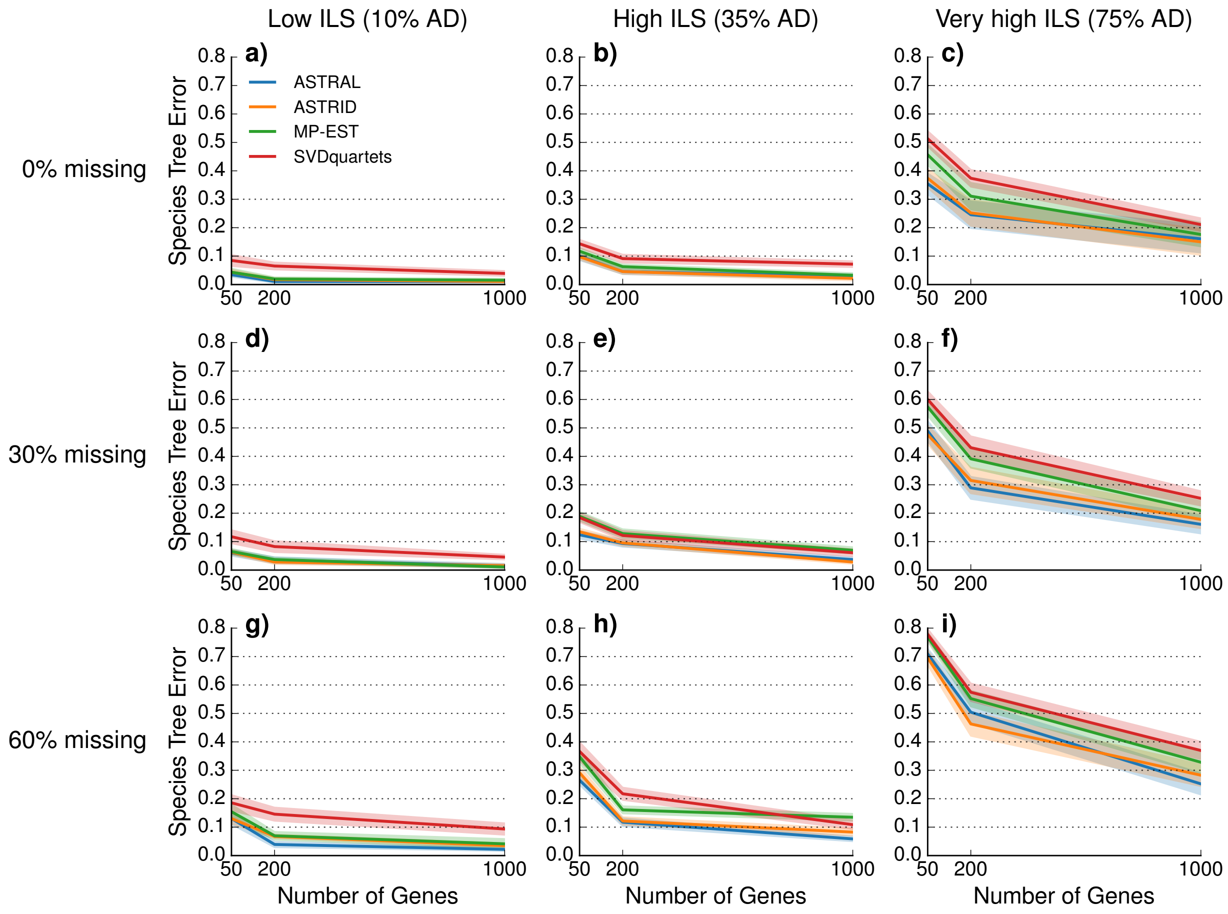

Figure S7: **Comparison of species tree estimation methods given estimated gene trees for the M_{iid} model of missing data (recent speciation).** Species tree error (measure by the RF error rate) is shown for species trees estimated by giving increasing numbers of genes as inputs to ASTRAL (blue), ASTRID (orange), MP-EST (green), and SVDquartets (red). All three summary methods were given **estimated gene trees**, and SVDquartets was given the true sequence alignment. Each column represents a different level of incomplete lineage sorting (ILS), and each row represents a different percentage of missing data. Lines represent the average over 20 replicate datasets, and filled regions indicate the standard error. Datasets shown here had recent speciation events; results for datasets with deep speciation events are shown in the main paper.

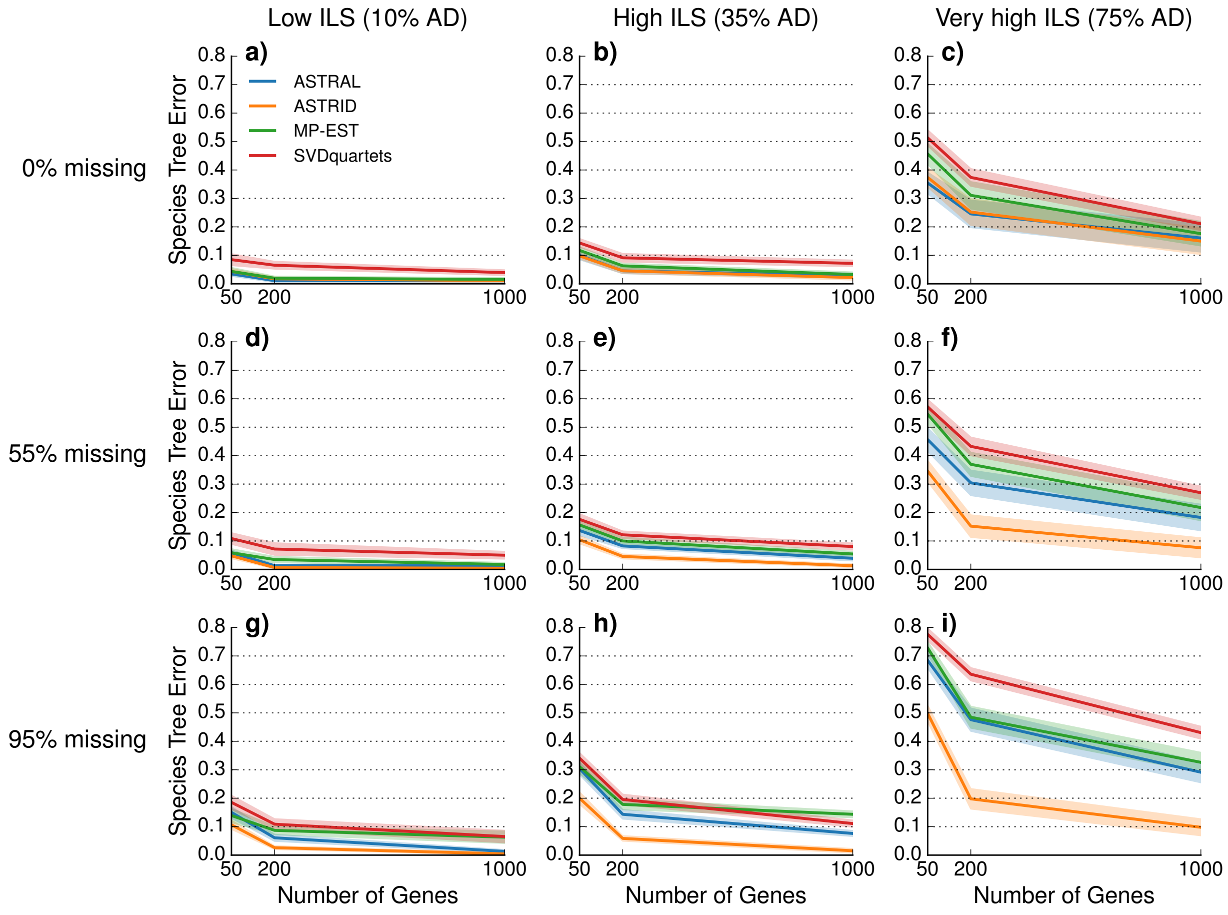

Figure S8: **Comparison of species tree estimation methods given estimated gene trees for the M_{clade} model of missing data (recent speciation).** Species tree error (measured by the RF error rate) is shown for species trees estimated by giving increasing numbers of genes as inputs to ASTRAL (blue), ASTRID (orange), MP-EST (green), and SVDquartets (red). All three summary methods were given **estimated gene trees**, and SVDquartets was given the true sequence alignment. Each column represents a different level of incomplete lineage sorting (ILS), and each row represents a different percentage of genes being incomplete genes due to clade-based missing data; for example, if 55% of the 1000 genes are incomplete, then 450 genes are complete and 550 genes are incomplete. Lines represent the average over 20 replicate datasets, and filled regions indicate the standard error. Datasets shown here had recent speciation events; results for datasets with deep speciation events are shown in the main paper.

References

Chifman, J. and L. Kubatko. 2014. Quartet Inference from SNP Data Under the Coalescent Model. Bioinformatics 30:3317–3324.

Chifman, J. and L. Kubatko. 2015. Identifiability of the unrooted species tree topology under the coalescent model with time-reversible substitution processes, site-specific rate variation, and invariable sites. Journal of Theoretical Biology 374:35–47.

Liu, L., L. Yu, and S. V. Edwards. 2010. A maximum pseudo-likelihood approach for estimating species trees under the coalescent model. BMC Evolutionary Biology 10:1–18.

Mirarab, S. and T. Warnow. 2015. ASTRAL-II: coalescent-based species tree estimation with many hundreds of taxa and thousands of genes. Bioinformatics 31:i44–i52.

Robinson, D. and L. Foulds. 1981. Comparison of phylogenetic trees. Mathematical Biosciences 53:131–147.

Sukumaran, J. and M. T. Holder. 2010. DendroPy: a Python library for phylogenetic computing. Bioinformatics 26:1569–1571.

Swofford, D. L. 2003. PAUP*. Phylogenetic Analysis Using Parsimony (*and Other Methods). Version 4. Sinauer Associates, Sunderland, Massachusetts.

Vachaspati, P. and T. Warnow. 2015. ASTRID: Accurate Species TRees from Internode Distances. BMC Genomics 16:1–13.
